# Desirable L-asparaginases for treating cancer and current research trends

**DOI:** 10.3389/fmicb.2024.1269282

**Published:** 2024-03-13

**Authors:** Kindu Tsegaye, Berhanu Andualem Tsehai, Birhan Getie

**Affiliations:** ^1^Department of Industrial Biotechnology, Institute of Biotechnology, University of Gondar, Gondar, Ethiopia; ^2^Department of Biotechnology, University of Gondar, Gondar, Ethiopia; ^3^Department of Industrial Biotechnology, Institute of Biotechnology, University of Gondar, Gondar, Ethiopia

**Keywords:** acute lympho blastic leukemia, cancer, kinetic properties, L-asparaginase, Michaelis constant

## Abstract

Amino acid depletion therapy is a promising approach for cancer treatment. It exploits the differences in the metabolic processes between healthy and cancerous cells. Certain microbial enzymes induce cancer cell apoptosis by removing essential amino acids. L-asparaginase is an enzyme approved by the FDA for the treatment of acute lymphoblastic leukemia. The enzymes currently employed in clinics come from two different sources: *Escherichia coli* and *Erwinia chrysanthemi*. Nevertheless, the search for improved enzymes and other sources continues because of several factors, including immunogenicity, *in vivo* instability, and protease degradation. Before determining whether L-asparaginase is clinically useful, research should consider the Michaelis constant, turnover number, and maximal velocity. The identification of L-asparaginase from microbial sources has been the subject of various studies. The primary goals of this review are to explore the most current approaches used in the search for therapeutically useful L-asparaginases and to establish whether these investigations identified the crucial characteristics of L-asparaginases before declaring their therapeutic potential.

## Introduction

Amino acid deprivation is an approach that shows promise for the development of novel cancer therapies. This natural remedy relies on the difference in metabolic processes between healthy and cancerous cells (Dhankhar et al., 2020). Because of their rapid growth, cancer cells produce fewer enzymes overall, which leads to auxotrophy for a subset of amino acids and makes them a target for enzymes that deplete amino acids. Therapeutic enzymes can only suppress tumor cells by cutting off amino acids, since normal cells can make their own. Amino acid deprivation therapy requires the use of certain enzymes, such as L-asparaginase, arginine deiminase, methionine, lysine oxidase, glutaminase, and phenylalanine ammonia-lyase (Dhankhar et al., 2020). L-asparaginases were first discovered as anticancer enzymes in 1922 (El-fakharany et al., 2020). The 22nd World Health Organization List of Essential Medicines now includes L-asparaginase as a cytotoxic therapy for acute lymphoblastic leukemia (WHO, 2021).

Leukemia is usually a cancer that causes an overabundance of aberrant white blood cells (blasts) in the bone marrow. Lymphoblastic leukemia affects lymphocytes that make up the lymph tissues 4. Acute lymphoblastic leukemia (ALL) is characterized by unchecked lymphocyte immature growth. L-asparaginase is one of the most widely used medications for treating ALL (Díaz-Barriga et al., 2021).

Antineoplastic drug successfully treated acute lymphoblastic leukemia (Radadiya et al., 2020; Sobat et al., 2020). L-asparaginase converts L-asparagine into aspartic acid and ammonia. When administered systemically, L-asparaginase (L-ASNase) reduces the availability of L-asparagine and prevents cancer cells from multiplying quickly and with a prime need for exogenous asparagine (van Trimpont et al., 2022). Lymphoblasts do not express asparagine synthetase (ASNS); hence, these cells must take extracellular asparagine to survive.

The L-asparaginases from *Escherichia coli* and *Erwinia chrysanthemi* have been used extensively in medicine, but growing complications like hypersensitivity, antigenicity, short half-life, temporary blood clearance, and unfavorable L-glutaminase-dependent neurotoxicity require ongoing research to find more suitable alternatives (Nguyen et al., 2018; Radadiya et al., 2020; Sobat et al., 2020). Problems with commercial L-ASNases have prompted research into better sources of the enzyme. This article summarizes the bacterial and fungal based L-asparaginases that are medically significant.

## L-asparaginases in the modern clinics

Today’s clinics use five distinct L-ASNase preparations (van Trimpont et al., 2022). The two ansB gene products from *E. chrysanthemi* are in the market under the trade names Erwinase and Rylaze. The ansB gene from *E. coli* was the source of the remaining three. Two of the three type II *E. coli* ASNases are stabilized formulations produced by the covalent conjugation of monomethoxy polyethylene glycol (PEG) to lysine on the enzyme via a succinimidyl succinate linker (Oncaspar) or succinimidyl carbonate linker (Asparlas). One of the three type II *E. coli* ASNases is a native formulation (Elspar and Kidrol). The addition of a PEG tag to L-ASNases prolongs their half-life and delays their removal from the body (van Trimpont et al., 2022).

The primary treatment for most ALL patients is PEG-native *E. coli* L-ASNase (EcA); unfortunately, many patients experience hypersensitivity reactions. This hypersensitivity may result from either a negative immunological response to the enzyme or the formation of neutralizing antibodies (often known as silent inactivation; Modi and Gervais, 2022). Native *E. chrysanthemi* L-ASNase (ErA) differs immunologically from PEG-native EcA and does not react with PEG-nEcA-derived antibodies. Therefore, patients who acquired an allergy to PEG-native EcA were administered native ErA (or, starting in July 2021, recombinant ErA) as second-line treatment (Modi and Gervais, 2022).

## L-asparaginases important in clinics

Type-I L-asparaginase, which has a low affinity for asparagine, is encoded by ansA in *E. coli*, whereas type-II L-asparaginase, which has a higher affinity for asparagine, is encoded by ansB. Acute lymphoblastic leukemia treatment employs type-II enzymes (Vimal and Kumar, 2017). The activity of an enzyme is often determined by adding a substrate to L-asparaginase and quantifying the amount of the output produced. While this measurement is a useful indicator for comparing activity between multiple enzymes within the same study, it is not always possible to compare this type of measurement to those from other studies. It is challenging to compare results from different research organizations because experiment-to-experiment variations in temperature, pH, and substrate quantity exist. In order to truly understand the therapeutic potential of a novel L-asparaginase, the kinetic parameters offer better insights (Beckett and Gervais, 2019).

The kinetic parameters provide insight into substrate affinity and turnover, which are unconditional values that can be consistently compared between study groups, and are essential for fully appreciating the therapeutic potential of a novel L-asparaginase. Before making claims regarding the therapeutic potential of a novel enzyme, Beckett and Gervais (2019) state that, at the very least, Michaelis constant (K_m_), turnover number (K_cat_), and maximum velocity (V_max_) should be established. However, many of the articles published in recent years lack this information (Abdelrazek et al., 2019; Kumar et al., 2019; Moguel et al., 2020; Prakash et al., 2020; Chakraborty and Shivakumar, 2021). In this review, few L-asparaginases from bacterial and fungal origin are reviewed, along with their corresponding kinetic properties and other characteristics.

## Michaelis constant (K_m_ value)

One requirement for the therapeutic effectiveness of L-asparaginases is the K_m_ value. Michaelis constant (K_m_) is the amount of a substrate at which the reaction rate is half of the maximum reaction rate. A lower K_m_ value shows that the enzyme is more active toward that substrate and can perform at half its maximum rate at lower substrate concentrations (Baral et al., 2020). For L-asparagine to completely disappear from circulation, a micromolar K_m_ is needed.

The K_m_ value is a quantifiable measure of the enzyme’s affinity for a substrate; the lower the K_m_ value, the greater the affinity of the enzyme for that substrate, and, in a therapeutic setting, the more effective the enzyme (Beckett and Gervais, 2019; Baral et al., 2020). Because intravenous L-asparaginase must be able to remove circulating pools of L-asparagine, which are not high (approximately 50 M), a low K_m_ (at least micromolar) of L-asparaginase is necessary (Loch and Jaskolski, 2021).

## Turnover number (K_cat_)

Turnover number, often known as K_cat_, is another helpful indicator for assessing enzyme activity. It provides the number of substrates that a single enzyme molecule converts into a product per unit of time (Baral et al., 2020). The substrate fully occupied the catalytic sites of the enzyme at the maximum reaction speed (V_max_). V_max_ is the amount of substrate that an enzyme can convert into a product in a second. The turnover number (K_cat_) is therefore the V_max_ divided by the enzyme concentration. The rate of transforming substrates into products increases as K_cat_ increases. A good therapeutic L-asparaginase must have a low K_m_ value and a high K_cat_ value to lower endogenous asparagine levels from 40 to 80 M to 0.02 M (Beckett and Gervais, 2019). Oncaspar and Erwinase, the two L-asparaginases that are currently on the market, have asparagine K_m_ and K_cat_ values of roughly 0.05 mM and 200–560 s^−1^, respectively (Beckett and Gervais, 2019; Baral et al., 2020).

## Glutaminase coactivity

The literature contains conflicting information regarding the secondary glutaminase activity of L-ASNases. From the FDA (United States)-approved *E. chrysanthemi* L-asparaginase, Nguyen et al. (2018) created a novel low glutaminase enzyme that was highly effective against T and B cell ALL (Nguyen et al., 2018). However, Chan et al. (2019) examined both the wild-type ASNase and its glutaminase-deficient mutant. They discovered that L-ASNase glutaminase activity, rather than ASNase activity alone, contributed to substantial anticancer efficacy against xenografts of the ASNS-negative leukemia cell line in NSG mice (Chan et al., 2019). This finding implies that, even against ASNS-negative cancer types, long-lasting, single-agent anticancer efficacy *in vivo* requires ASNase glutaminase activity. Horvath et al. (2019) investigated the pharmacodynamics of ASNase in a mouse model of cancer. They showed that asparagine was detected after L-ASNase treatment, in contrast to previously published methods that produced post-ASNase asparagine levels below the detection threshold (Horvath et al., 2019). They ascribed these differences to the use of whole blood, which collects the target analyte in both the red blood cell and plasma, as opposed to only serum or plasma.

A different study by Nguyen et al. (2017) suggested that a single-point mutation controls the substrate selection of L-asparaginases. They claimed that whereas the L-glutaminase activities of *Wolinella succinogenes* L-asparaginase (WoA) with proline at position 121 (WoA-P121) and WoA with serine at position 121 (WoA-S121) were noticeably different, their kinetic characteristics were identical. L-glutaminase activity is present in the WoA variant with a proline at position 121 but not in WoA-S121 (Nguyen et al., 2017). Nguyen et al. (2017) concluded that the residue at position 121 controls Asn vs. Gln selectivity via a mechanism other than substrate binding, which calls for further research.

## Toxicity

L-asparaginase’s toxicity is caused by either a secondary L-glutaminase activity or a hypersensitivity to the foreign protein (Baral et al., 2020; Fonseca et al., 2021; van Trimpont et al., 2022). When the body perceives an enzyme as foreign, it triggers an immunological reaction that results in a minor allergic reaction to anaphylactic shock (Baral et al., 2020). Glutaminase coactivity results in the hydroxylation of glutamine to glutamate and ammonia. Excessive ammonia generation may cause neurotoxicity and liver malfunction (van Trimpont et al., 2022). Ashok et al. (2019) eliminated toxicity caused by *E. coli* and *E. chrysanthemi* L-ASNases by purifying the urease (Ashok et al., 2019). Urease catalyzes the hydrolysis of urea, which results in the production of ammonia and carbon dioxide. As a result, ammonia is excreted from the bloodstream as urea is converted back to ammonia, resulting in hyperammonemia, a condition that damages the brain (Ashok et al., 2019). Chakraborty and Shivakumar (2021) also described a glutaminase and urease-free L-asparaginase from *Agaricomycete* sp. *Ganoderma australe* GPC191 (Chakraborty and Shivakumar, 2021). The urease activity of L-ASNases was not, however, reported in many of the studies.

## The need for exogenous sources of L-asparaginases

Humans possess a type III L-asparaginase that needs a threonine residue at the N-terminus to catalyze reactions. Unfortunately, because of its millimolar K_m_ value for L-asparagine, natural human L-asparaginase is not suitable for therapeutic uses. L-asparaginases must have a micromolar K_m_ for asparagine in order to remove it from the blood (Rigouin et al., 2017). To get rid of the circulating L-asparagine in ALL patients, type II L-asparaginases from *E. coli* or *E. chrysanthemi* are utilized (Radadiya et al., 2020; Andrade et al., 2021). Wild type II L-asparaginases exhibit high selectivity for L-asparagine and low glutaminase side activity (2%–10%).

## Kinetic parameters of microbial L-asparaginases in recent studies

L-asparaginase is a therapeutic enzyme used to treat cancer. The major obstacles to the therapeutic potential of this enzyme are immunogenicity, short plasma half-life, and glutaminase activity (Vidya et al., 2017). Several research and development projects are underway to address these problems. To identify a novel therapeutic L-asparaginase, studies must consider V_max_, K_m_, and K_cat_ values comparable to or superior to those of current therapeutic therapies (Beckett and Gervais, 2019). Plants, animals, and microbes naturally produce L-asparaginases. Compared to plant and animal sources, microbes are more cost-effective and easier to modify, optimize, extract, and purify for enzyme synthesis (Fazeli et al., 2021). In addition, genetically altering microbes to boost yields is simple (Costa-Silva et al., 2019). Because bacterial and fungal L-asparaginases are thought to be important in both medicine and industry, this review focuses on their kinetic properties.

## Kinetic parameters of bacterial L-asparaginases

Although other bacterial species can produce L-asparaginases, only type-II L-asparaginases from *E. coli* and *E. chrysanthemi* have been produced on an industrial scale (Vimal and Kumar, 2017). There are two forms of bacterial L-asparaginases: intracellularly produced type I, or cytosolic, and extracellularly created type II, or periplasmic (El-fakharany et al., 2020). Most bacteria produce type I, or cytosolic, L-asparaginases within their cells, but a few secrets type II, or periplasmic, L-asparaginases outside. Since gram-positive bacteria do not have a periplasmic gap, they secrete much more enzymes into the environment than gram-negative bacteria (Vimal and Kumar, 2017).

The production of extracellularly secreted enzymes is more beneficial for industrial processes because these can be made in large quantities in a culture of ideal conditions, and it takes less money and effort to purify them (Vimal and Kumar, 2017; El-fakharany et al., 2020). As a result, it would be more profitable to test for extracellular enzymes in gram-positive bacteria. The type II bacterial L-asparaginases are the focus of this review since they are crucial for clinical outcomes. Table 1 shows bacterial strains that produce L-ASNase, along with their characteristics. The review includes studies from 2017 to present and reported the kinetic property of L-ASNases. According to the data in Table 1, *Bacillus velezensis*, *Streptomyces brollosae* NEAE-115, and *Bacillus halotolerans* OHEM18 have low K_m_ values as compared to commercial L-ASNases, signifying that they might be potential candidates. L-ASNase from *Bacillus halotolerans* OHEM18 also showed an antioxidant activity against 2, 2′-azino-bis [3-ethylbenzothiazoline-6-sulfonic acid (ABTS) and 2, 2′-diphenyl-1-picrylhydrazyl (DPPH) radicals (Table 1)].

**Table 1:** Kinetic parameters and other features of bacterial type-II L-asparaginase.

Bacterial species	K_m_ for Asn	V_max_ for Asn	Spec. activity U mg^−1^	Half-life or stability	Other properties	Source	Effect on	Ref.
*Bacillus altitudinis*	9.09 × 10^−2^ M	0.09 Ms^−1^	800	Opt. 37°C	Glutaminase free	Soil	Leukemia	Prakash et al. (2019)
*Bacillus velezensis*	3.6×10^−5^ M	41.49 μmol mL^−1^ min^−1^	31.77	Opt. 37°C	Glutaminase free	Marine sediments	Breast cancer	Mostafa et al. (2019)
*Bacillus licheniformis*	0.049995 M	45.45 μmol mL^−1^ min^−1^	36.08	Stable at 70°C for 1 h	Glutaminase free Stabile at 70°C for 1 h	Water Red sea	Breast, colon HepG2 live	Alrumman et al. (2019)
*Streptomyces brollosae NEAE-115*	2.139 × 10^−3^ M	152.6 UmL^−1^ min^−1^	76.671	65.02 min at 50^0^С	Glutaminase free extracellular Reduced immunogenicity	Soil	Ehrlich Ascites Carcinoma	El-naggar et al. (2018)
*Bacillus halotolerans OHEM18*	0.0047 M	92.74Uml^−1^ min^−1^	215.33	70% activity for 1 h at 50°C	Extracellular antioxidant against DPPH^*^ and ABTS^**^ radicals	Soil	Leukemia, breast, hepatoma cells	El-fakharany et al. (2020)
*Streptomyces* sp.	0.065 mM	20.80 IU ml^−1^	390 IU mg^−1^	Stable till 50°C	Thermostable at 50°C	Soil	Not reported	Desai and Hungund (2018)

^*^DPPH, 2, 2 diphenyl-1-picrylhydrazyl. ^**^ABTS, 2, 2′-azino-bis (3-ethylbenzothiazoline-6-sulfonic acid).

## Kinetic parameters of fungi-derived L-asparaginases

Bacterial sources of L-asparaginase have been linked to a variety of immunological reactions, including hypersensitivity, irregular clotting, and allergic reactions. To reduce these immunological issues, a different supply is required. Fungi have evolved more closely with humans than bacteria, so it is likely that the enzymes they produce will have lower immunogenicity than bacteria (Ashok et al., 2019; Costa-Silva et al., 2019).

It has been claimed that a different source of L-asparaginases with fewer negative effects comes from fungi. Fungal species can be employed more successfully than other microbes to cure cancer because of their eukaryotic origin and their ability to reproduce the actions of human cells (Ashok et al., 2019). There may be fewer negative effects because yeast ASNase is more similar to human congeners (Karla et al., 2020). A variety of yeast genera, including *Candida, Pichia, Rhodosporidium, Saccharomyces*, and *Yarrowia*, have been reported to generate ASNase (Karla et al., 2020). Owing to appropriate post-translational protein modification and glycosylation, yeasts are good eukaryotic candidates for the production of asparaginase (Darvishi et al., 2018). The kinetic characteristics of L-asparaginases from fungi, as reported in recent literature, are shown in Table 2. According to Table 2, *Aspergillus terreus*, a marine sediment isolate, had a significantly high K_m_ compared to *E. coli* and *E. chrysanthemi*. Due to its kinetic characteristics, K_m_ = 9.37 M, V_max_ = 127 M/ML/min, and specific activity of 468.03 U/mg, the plant parasitic fungus *Lasiodiplodia theobromae* appears to be a suitable candidate for L-ASNase synthesis (Table 3).

**Table 2:** The table lists recent studies that include recombinant microbial L-asparaginases and their accompanying characteristics.

Original microbe	Source	Gene	Host	K_m_	V_max_	Half-life or stability	Other properties	Ref.
*Bacillus* sp. *SL-1*	Salt lake	ansA1	*E. coli* BL21	10.30 μM	Not reported K_cat_ 23.96 s^−1^	Stable at −20°C for 1 year	−Glutaminase pH range 4.5–10	Safary et al. (2019)
*Cobetia amphilecti AMI6*	Mangrove (shore line plant) sediments	CobAsnase AMI6 gene	*E. coli*	2.05 mM	11,641 μM min^−1^ mg^−1^	68.26% activity after 60 min at 50°C	No detectable glutamine activity	Farahat et al. (2020)
*Melioribacter roseus*	Oil exploration well—Russia	L-ASNase-MrAIII Plant type	*E. coli BL21(DE3)*	1.4 mM	5,573 μM min^−1^	70% activity after 1 h at 40°C	+Glutaminase (19%)	Dumina et al. (2021a)
*Thermococcus kodakarensis*	Thermophilic archaea	TK2246 gene^*^	*E. coli BL21(DE3)*	3.1 mM	833μmol mg^−1^ min^−1^		Thermostable −80°C, Negligible glutaminase	Chohan et al. (2020)
*Pyrococcus furiosus*	NCBI CP003685.1	L-ASNase gene Uniprot Q8U4E6	*E. coli BL21(DE3)*	1.623 mM	105 μmol min^−1^ mg^−1^	98.3% activity after 1 h at 37°C	Glutaminase free Urease free Shelf-life—56 days at 4°C	Saeed et al. (2020)
*Bacillus subtilis strain R5*	Culture from Osaka	Asn-R5 gene	*E. coli.*	2.4 mM	265 μmol min^−1^ mg^−1^,	180 min at 35°C	low glutaminase activities, thermal stability	Chohan and Rashid (2018)
*Pseudomonas fluorescens MTCC 8127*	Culture collection	ANS gene	*E. coli BL21 (DE3)*	0.050 M	4.032 mol min^−1^	40 h	Minimal Glutaminase activity	Sindhu and Manonmani (2017)
*Lactobacillus casei subsp. casei ATCC 393*	Culture collection	ansB gene	*E. coli*	0.01235 mM	1.576 mM/min	serum -44 h trypsin—15 min	Active from 10 to 80°C	Aishwarya et al. (2019)
*Bacillus tequilensis PV9W*	Gene bank KR261609	ansA	*E. coli BL21 (DE3)*	0.04 mM	10.21 μmol mL^−1^ min^−1^	Trypsin-120 min	Cervical cancer stable for 25 days at 25°C, least immunogenicity	Shakambari et al. (2018)
*Halomonas elongata*	Culture collection Iran	L-ASNase gene	*E. coli BL21 (DE3)*	5.6 mM	2.2 μmol min^−1^, K_cat_ 1.96 × 10^3^ s^−1^	Serum-90 min	L-glutaminase activity-1%	Ghasemi et al. (2017)
*Lactobacillus reuteri DSM 20016*	Culture collection	ORF LREU_RS09880	*E. coli BL21(DE3)*	0.3332 mM	14.06 mM min^−1^	human serum-44 h trypsin—15 min	leukemic cell lines	Aishwarya et al. (2017)
*Bacillus sonorensis*	Soil	L-ASNase gene	*E. coli BL21(DE3)*	2.004 mM	3,723 μmol min^−1^	Retain 92% activity at 37°C	No urease activity	Aly et al. (2020)
*Pyrobaculum calidifontis-Archaea*	NCBI CP000561.1	Pcal_0970 gene	*E. coli BL21-CodonPlus (DE3)-RIL*	4.5 mmol/L	355 μmol min^−1^ mg^−1^	150 min at 100°C	Undetectable Glutaminase activity	Chohan et al. (2018)
*Thermococcus sibiricus*	Oil reservoir Serbia	*tsA_wt Gene bank WP_015849943.1*	*E. coli BL21 (DE3)*	2.8 mM	1,200 μM min^−1^	86% activity after 20 min at 90°C	Low glutaminase activity	Dumina et al. (2021b)

**Table 3:** Features of fungal L-asparaginases in current research articles

Fungal/yeast species	K_m_	V_max_	Spec. activity	Half-life or stability	Other properties	Source	Ref.
*Lasiodiplodia theobromae*	9.37 μM	127.00 μM mL^-1^min^-1^	468.03 U/mg	Stable at 37 ^0^C for 4 h	Extracellular	Culture collection-Egypt	Moubasher et al. (2022)
*A.oryzae CCT 3940*	2.10 mM	35.8 U mL^−1^	Note reported	Stable after 60 min at 50^0^C	Glutaminase free	Culture collections (Brazil)	Dias et al. (2019)
*A.oryzae LBA 01*	5.07 mM	57.14 U mL^−1^	Note reported	Only 60% after 60 min at 50 °C	Glutaminase free		
*A. niger LBA 02*	1.41 mM	39.22 U mL^−1^	Note reported	stable after 60 min at 50^0^C	Low Glutaminase activity		
*Aspergillus terreus*	31.5 mM	500U mL^−1^	268.5U Mg^−1^	2826.90 min at 50^°^C	Glutaminase activity absent	Marine environment	Hassan et al. (2018)
*Sarocladium strictum (yeast)*	9.74 mM	8.19 μmol min^−1^	Note reported	Note reported	Glutaminase free Mostly extracellular	Soil	Golbabaie et al. (2020)
*Fusarium equiseti AHMF4*	Not reported	Not reported	488.1 Umg^−1^	100% activity for 1 h from 20^°^C to 40^°^C	Antioxidant—DPPH^*^ anti-proliferative—cervical, hepatocellular, colorectal, breast	Soil	El-Gendy et al. (2021)
*A. oryzae IOC 3999*	3.28 mMol L^−1^	45.04 U mL^−1^	742.22 Umg^−1^ K_cat_ 0.93s^−1^	Stable at 50^o^C for 1 h	Not reported	Culture collection-FioCruz	Da Cunha et al. (2021)
*Sarocladium kiliense*	0.025 mM	0.30 μmol ml^−1^min^-1^	919	Stable in human serum for48 h	Not reported	Marine water	Bhargavi and Madhuri (2021)

^*^DPPH, 2, 2 diphenyl-1-picrylhydrazyl.

## The search for L-asparaginase is still ongoing

The *E. chrysanthemi* (ErAII) enzyme is only utilized when there are immunological reactions to *E. coli*-derived ASNases, and *E. coli* (EcAII) type II ASNase, whether in its native or PEGylated form, is currently the medication of choice. However, these L-ASNases have a high degree of *in vivo* instability, a short half-life, and require many administrations to be therapeutically effective (Maggi et al., 2017). Additionally, the immunological response, proteolytic degradation, and toxic side effects of type II bacterial L-ASNases limit their clinical usage (Radadiya et al., 2020). Numerous trials have been conducted worldwide in an attempt to reduce these problems.

Different strategies have been used to produce L-asparaginases with the best qualities for medicinal use. Recombinant ASNase synthesis, enzyme encapsulation, structural alteration of the enzyme, and the hunt for new ASNase-producing microbes are some of these methods (Sobat et al., 2020; Belén et al., 2021; Díaz-Barriga et al., 2021). L-asparaginase is found throughout all spheres of life; thus, a variety of sources, including bacteria, fungi, plants, and mammals, have been screened to identify enzymes with superior characteristics (Sobat et al., 2020).

## Finding new L-asparaginases from new sources

Extreme conditions, such as salinity or high temperature, which cause the inactivation of enzymes isolated from terrestrial microorganisms, are widely used to produce enzymatic operations on an industrial scale. The marine environment attracts special interest in the search for new sources for economically significant products due to its extraordinary diversity and harsh circumstances (Alrumman et al., 2019; Mostafa et al., 2019; Sharma et al., 2019; Sobat et al., 2020; Bhargavi and Madhuri, 2021). Bacteria that are halotolerant or halophilic often express and synthesize enzymes that can withstand extreme conditions, such as high temperatures, high salt concentrations, the presence of organic solvents, pH, and non-physiological values (Dumina et al., 2020; El-fakharany et al., 2020). Given that these enzymes are naturally exposed to large levels of osmolytes, such as NaCl or other suitable solutes, it would make sense to anticipate enhanced biological activity and/or osmolarity tolerance in physiological circumstances of blood serum (Ghasemi et al., 2017).

A new L-asparaginase from the hyperthermophilic archaeon *Thermococcus sibiricus* has been described by Dumina et al. (2021a). They cloned the L-ASNase gene of *Thermococcus sibiricus* into *E. coli* and studied its kinetics. The findings indicated a good contender, with K_m_ and V_max_ for the enzyme being 2.8 mM and 1,200 M/min, respectively (Dumina et al., 2021b). El-fakharany et al. (2020) discovered Bacillus halotolerans OHEM18, which can produce L-ASNases with K_m_ = 0.0047 M and V_max_ = 92.74 2. This strain makes an essential extracellular L-ASNase, which is crucial for industry. In addition, Chakravarty et al. (2021) found *Bacillus australimaris* NJB19 in oceanic sediments and cloned the ASNase genes into *E. coli* to produce enzyme (Chakravarty et al., 2021). They discovered that the enzyme was effective against leukemic cells and had high affinity for asparagine. Tables 2, 3 show the results of several other investigations into the search for novel L-ASNases.

## Reduced or absent co-activity in L-ASNase variants

The glutaminase co-activity of L-ASNase therapy has been implicated in the majority of its non-immune-related severe side effects. Thus, a decrease in glutaminase coactivity might significantly enhance the toxicity profile of L-ASNase. Alrumman et al. (2019) isolated *Bacillus licheniformis* from the Red Sea, which produces a glutaminase-free ASNase and may be a future candidate for pharmaceutical use as an anticancer medication *34*. Ashok et al. (2019) screened fungal species from Antarctic soil and moss that generate L-asparaginase devoid of glutaminase and urease (Ashok et al., 2019). Glutaminase-free L-ASNase has been tested in numerous investigations from various sources (El-naggar et al., 2018; Ashok et al., 2019; Mostafa et al., 2019; Safary et al., 2019; Karla et al., 2020; Prakash et al., 2020; Saeed et al., 2020; Prihanto et al., 2022). Further studies are necessary because there is still controversy regarding the coactivity of L-glutaminase asparaginase.

## Encapsulation

Encapsulation is a promising and innovative strategy to improve the *in vivo* performance of ASNase because it prevents the enzyme from coming into direct contact with the environment, shields it from protease degradation, extends the enzyme’s catalytic half-life, and, in some cases, lowers immunogenicity (Villanueva-flores et al., 2021; Guimarães et al., 2022). Shakambari et al. (2018) encapsulated L-ASNase from *Bacillus tequilensis* PV9W in solid lipid particles made from palmitic acid (Shakambari et al., 2018). In comparison to the natural L-asparaginase, the lipid particle-encapsulated version had increased V_max_ (7.790.34 to 10.211.43), a K_m_ that was cut in half (0.0700.01 to 0.040.001), a better half-life (50.19 to 120.56 min), and was stable for 25 days when stored at 25°C (Shakambari et al., 2018).

Encapsidation in a scaffold or substrate is another highly effective method to enhance the pharmacodynamics and pharmacokinetics of ASNase. ASNase II from *E. coli* was genetically engineered by Dáz-Barriga and colleagues into virus-like proteins of the bacteriophage P22. According to their findings, the encapsulated ASNase had a 15-fold and a 2-fold increase in K_m_ and K_cat_ compared to the unbound enzyme (Díaz-Barriga et al., 2021). These virus-like particles are made up of numerous copies of self-assembling proteins, but because they lack genetic material, they are unable to spread diseases. Their self-assembly abilities enable the development of a nanoparticle that is identical to the original viral capsid, except that it does not contain genetic material and instead includes an interesting chemical, in this example L-ASNase (Díaz-Barriga et al., 2021). The key findings, limitations, and existing knowledge gaps related to ASNase nano- and micro-encapsulation are well-reviewed in Villanueva-flores et al. (2021).

## Site-directed mutagenesis

Site-directed mutagenesis can be used to generate EcAII variants with improved characteristics (Maggi et al., 2017). The stability, immunogenicity, and substrate specificity of L-ASNases can be significantly enhanced by using this method. According to a study by Maggi et al. (2017), Asn24 plays a critical role in maintaining EcAII active sites. For proteases that break down, asn24 serves as a cleavage point. Maggi et al. created the EcAII N24S variant through site-directed mutagenesis, which maintains asparaginase and glutaminase functions while exhibiting long-term storage stability and protease resistance (Maggi et al., 2017).

## Structure-based rational design

Structure-based rational design is a potent protein engineering strategy to enhance the enzymatic capabilities of ASNases. Using bioinformatics servers (HotSpot Wizard and EVcoupling), rational design approach first screens active center residues using structural analysis, compares sequences, and identifies distal residues, allowing the construction of smart libraries for performing mutations at specific sites with less screening work (Zhou et al., 2022). Zhou et al. (2022) increased the type II ASNase from *Bacillus licheniformis’s* catalytic activity by using semi-rational design (Zhou et al., 2022). According to their findings, the mutant’s K_m_ value was 1.45 mM instead of 2.33 mM, and its K_cat_ value was 778.87 min-1 as opposed to 197.95 min^−1^ in the wild type. According to Aghaeepoor et al. (2018), the N248S mutation is linked to an appropriate L-ASNase function, while disrupting glutaminase activity due to hampered interactions. They stated that *in silico* analysis methods would provide useful information for creating mutant enzymes (Aghaeepoor et al., 2018). Yari et al. (2019) also described the significance of computational techniques for preliminary screening of appropriate mutations.

## Using *in silico* screening

Traditional approaches to drug discovery are mainly based on *in vitro* drug screening and *in vivo* animal experiments; however, these methods are usually expensive and laborious. In recent years, omics data have provided an opportunity for the computational prediction of anti-cancer drugs, improving the efficiency of drug discovery (Baral et al., 2020; Li et al., 2020). Over time, there has been significant improvement in bioinformatics databases and methods used for drug screening, structural design, and immunogenicity prediction. This offers an opportunity to find anticancer medications that are less expensive and time consuming. Li et al. (2020) reviewed the databases and computational tools available for creating new cancer treatment approaches.

The basis of enzyme catalysis is the binding energy, which decreases the activation energy and circumvents the adverse entropic conditions necessary for the correct alignment of the enzyme and its substrates. Free energy was used to measure the binding energy, which is the energy generated when a substrate forms a weak connection with an enzyme’s active site (Delta G). It is challenging to calculate this energy experimentally; therefore, an *in silico* method utilizing docking software would be more effective (Baral et al., 2020).

According to research by Baral et al. (2020), asnB genes from *Streptomyces griseus*, *Streptomyces venezuelae*, and *Streptomyces collinus* have higher binding energies than those from *E. coli* and *E. chrysanthemi* and were predicted to have the lowest Kms because binding energy and K_m_ are inversely correlate 20. As-Suhbani and Bhosale (2020) demonstrated in a different investigation that the interactions between the ligand L-asparagine and Fusarium solani CLR-36 L-asparaginase occurred at the active site with good binding energy (−6.85 kcal/mol; As-Suhbani and Bhosale, 2020).

Numerous studies have addressed the use of bioinformatics tools to analyze immune responses using *in silico* methods. Using the prediction tool EMBOSS antigenic explorer, Abdelrazek et al. (2019) identified possible antigenic areas of the L-asparaginase sequence of *Bacillus licheniformis* and compared them to those of *E. coli* and *E. chrysanthemi*. For the L-asparaginases of *E. coli*, *E. chrysanthemi,* and *Bacillus licheniformis*, 18, 16, and 17 antigenic areas, respectively, were discovered (Abdelrazek et al., 2019). Using a bioinformatics-based methodology, Belén et al. (2021) also predicted the immunogenicity of L-ASNases from nine filamentous fungi and compared the outcomes with those of *E. coli* L-ASNase (Belén et al., 2021).

## Using recombinant DNA technology

Although hypersensitivity reactions frequently prevent their use, bacterial L-asparaginase has long been a crucial component of ALL treatment. For individuals with hypersensitivity, alternative asparaginase preparations are required to guarantee asparaginase availability. Recombinant technology has the potential to fill this unmet need by engineering cells to produce recombinant asparaginase with higher recovery, lower immunogenicity, and cheaper production costs (Vimal and Kumar, 2017), as well as by enhancing the characteristics of numerous commercially significant enzymes (Shakambari et al., 2018). Maese et al. (2021) described a recombinant *Erwinia asparaginase* (JZP-458) with no immunological cross-reactivity with *E. coli*-derived asparaginases *in vitro*. It was derived from a new *Pseudomonas fluorescens* production platform. The US Food and Drug Administration approved JZP-458 (Rylaze) in June 2021 for the treatment of ALL in patients younger than 1 month old who exhibited hypersensitivity to asparaginase produced by *E. coli* (Maese et al., 2021). The process of cloning the L-asparaginase gene from an organism into *E. coli* has been attempted numerous times with success. Table 2 presents the results of a few of these attempts.

## Author contributions

KT: Conceptualization, Writing – original draft, Writing – review & editing, Methodology, Validation, Investigation. BT: Validation, Writing – review & editing, Conceptualization, Supervision. BG: Validation, Writing – review & editing, Methodology.
