# Supplementary material for: Desirable L-asparaginases for treating cancer and current research trends
Source: Front Microbiol. 2024 Mar 13;15:1269282. doi: 10.3389/fmicb.2024.1269282 (PMC11001194; doi:10.3389/fmicb.2024.1269282)
Supplement: Supplementary file 1 [file Presentation_1.PPTX]

## Slide 1
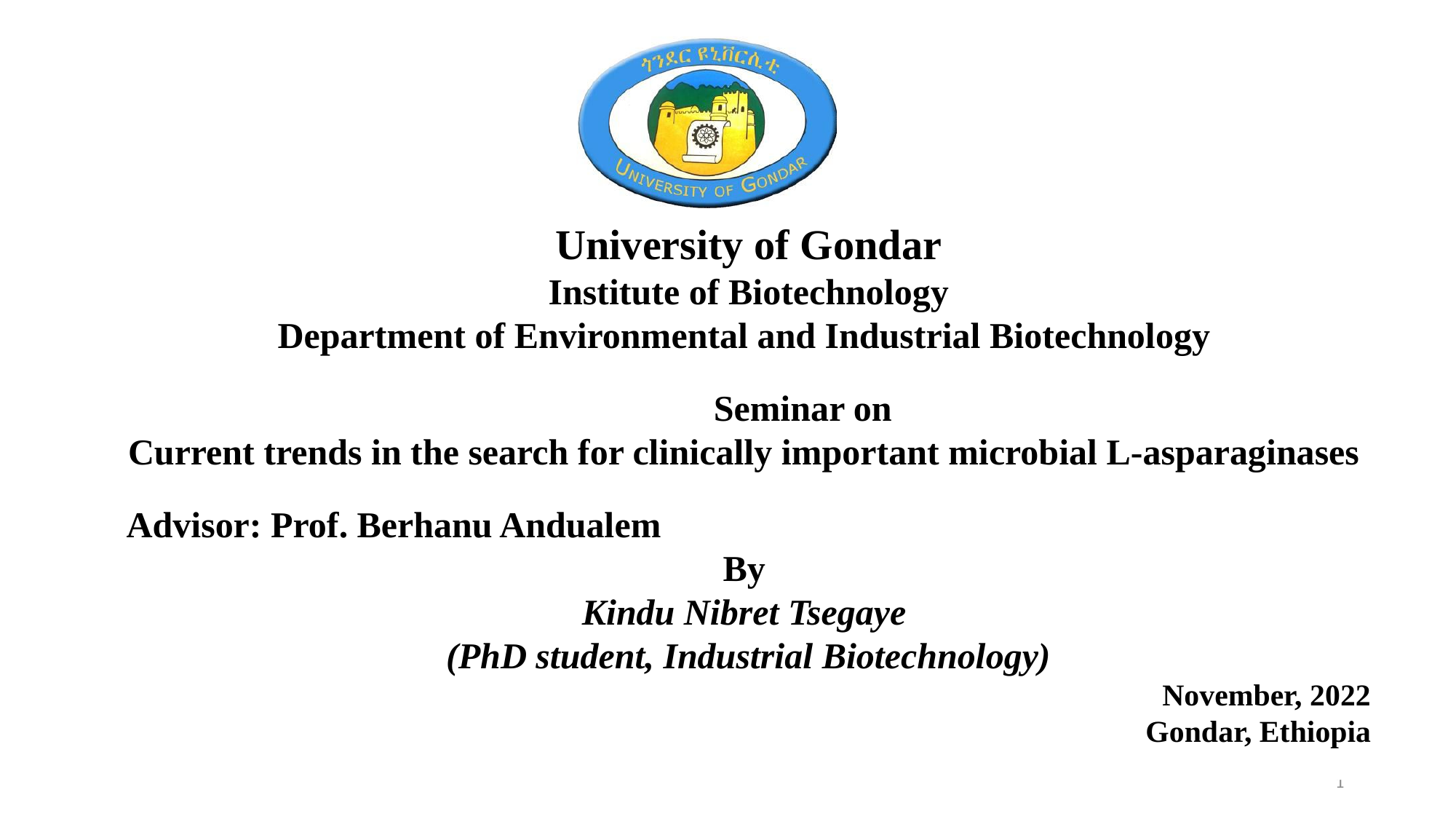

University of Gondar
Institute of Biotechnology
Department of Environmental and Industrial Biotechnology
 Seminar on
Current trends in the search for clinically important microbial L-asparaginases
Advisor: Prof. Berhanu Andualem
By
Kindu Nibret Tsegaye
(PhD student, Industrial Biotechnology)
November, 2022
Gondar, Ethiopia
1

## Slide 2
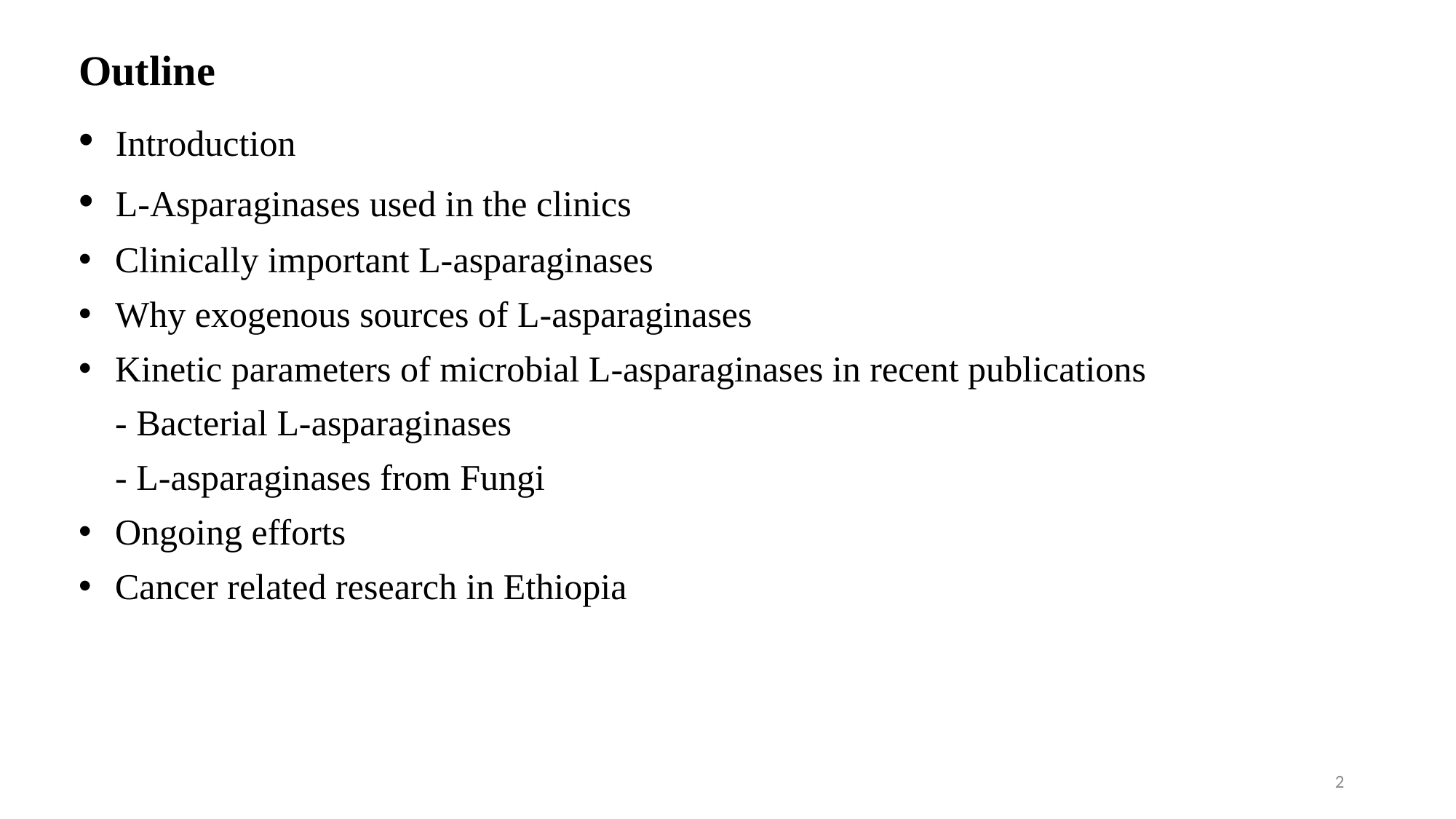

# Outline
 Introduction
 L-Asparaginases used in the clinics
 Clinically important L-asparaginases
 Why exogenous sources of L-asparaginases
 Kinetic parameters of microbial L-asparaginases in recent publications
 - Bacterial L-asparaginases
 - L-asparaginases from Fungi
 Ongoing efforts
 Cancer related research in Ethiopia
2

## Slide 3
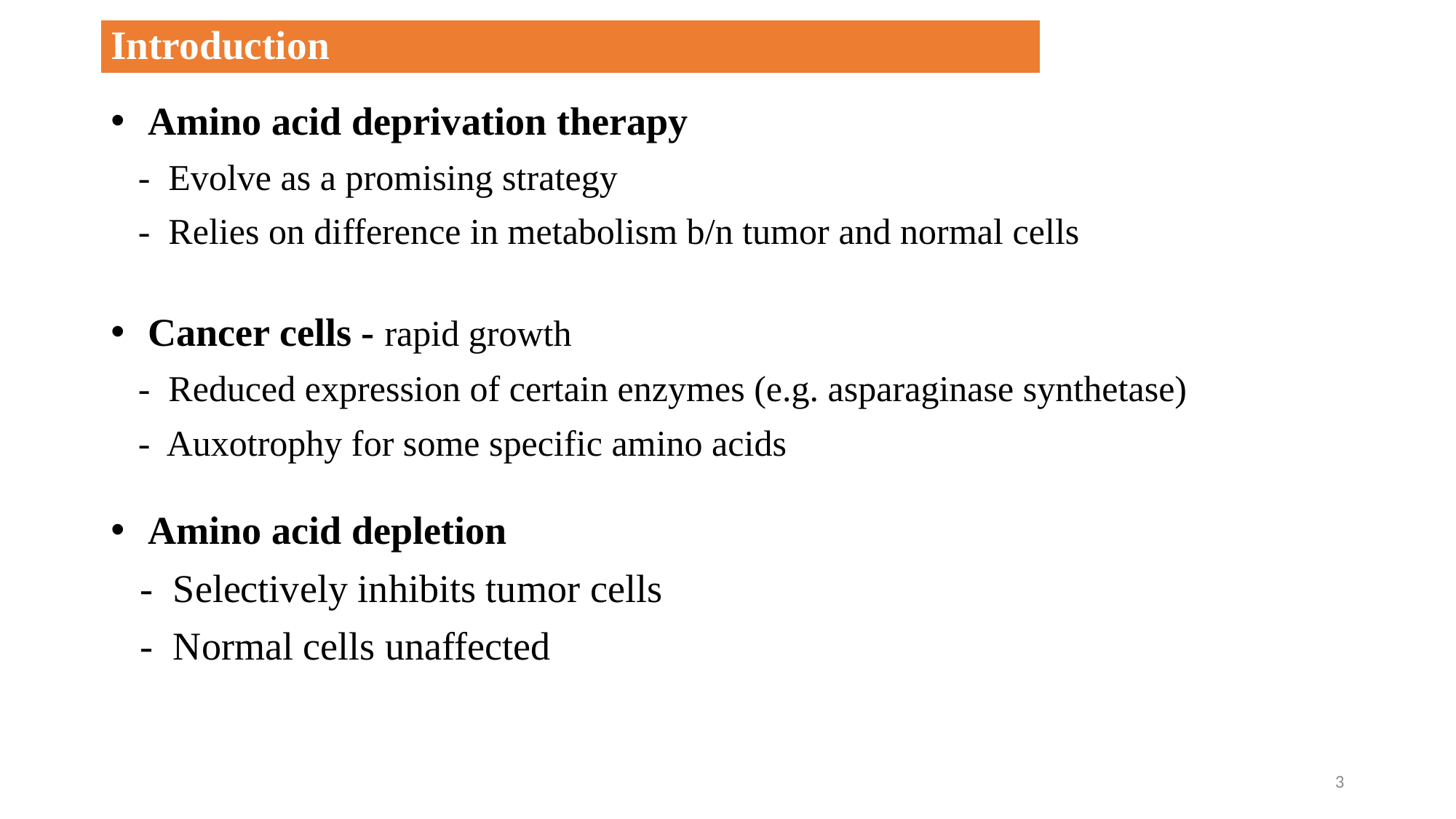

# Introduction
 Amino acid deprivation therapy
 - Evolve as a promising strategy
 - Relies on difference in metabolism b/n tumor and normal cells
 Cancer cells - rapid growth
 - Reduced expression of certain enzymes (e.g. asparaginase synthetase)
 - Auxotrophy for some specific amino acids
 Amino acid depletion
 - Selectively inhibits tumor cells
 - Normal cells unaffected
3

## Slide 4
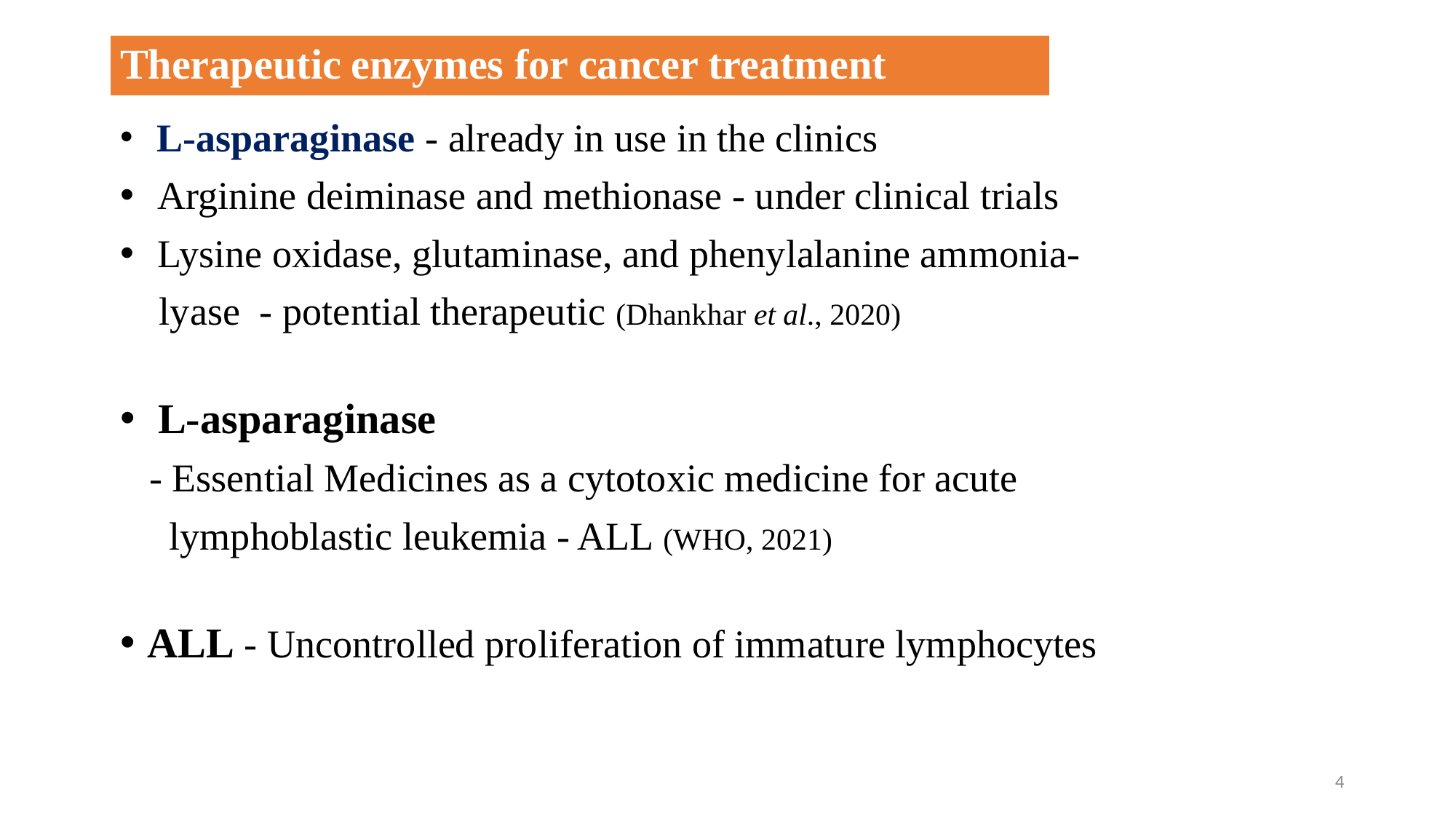

# Therapeutic enzymes for cancer treatment
 L-asparaginase - already in use in the clinics
 Arginine deiminase and methionase - under clinical trials
 Lysine oxidase, glutaminase, and phenylalanine ammonia-
 lyase - potential therapeutic (Dhankhar et al., 2020)
 L-asparaginase
 - Essential Medicines as a cytotoxic medicine for acute
 lymphoblastic leukemia - ALL (WHO, 2021)
ALL - Uncontrolled proliferation of immature lymphocytes
4

## Slide 5
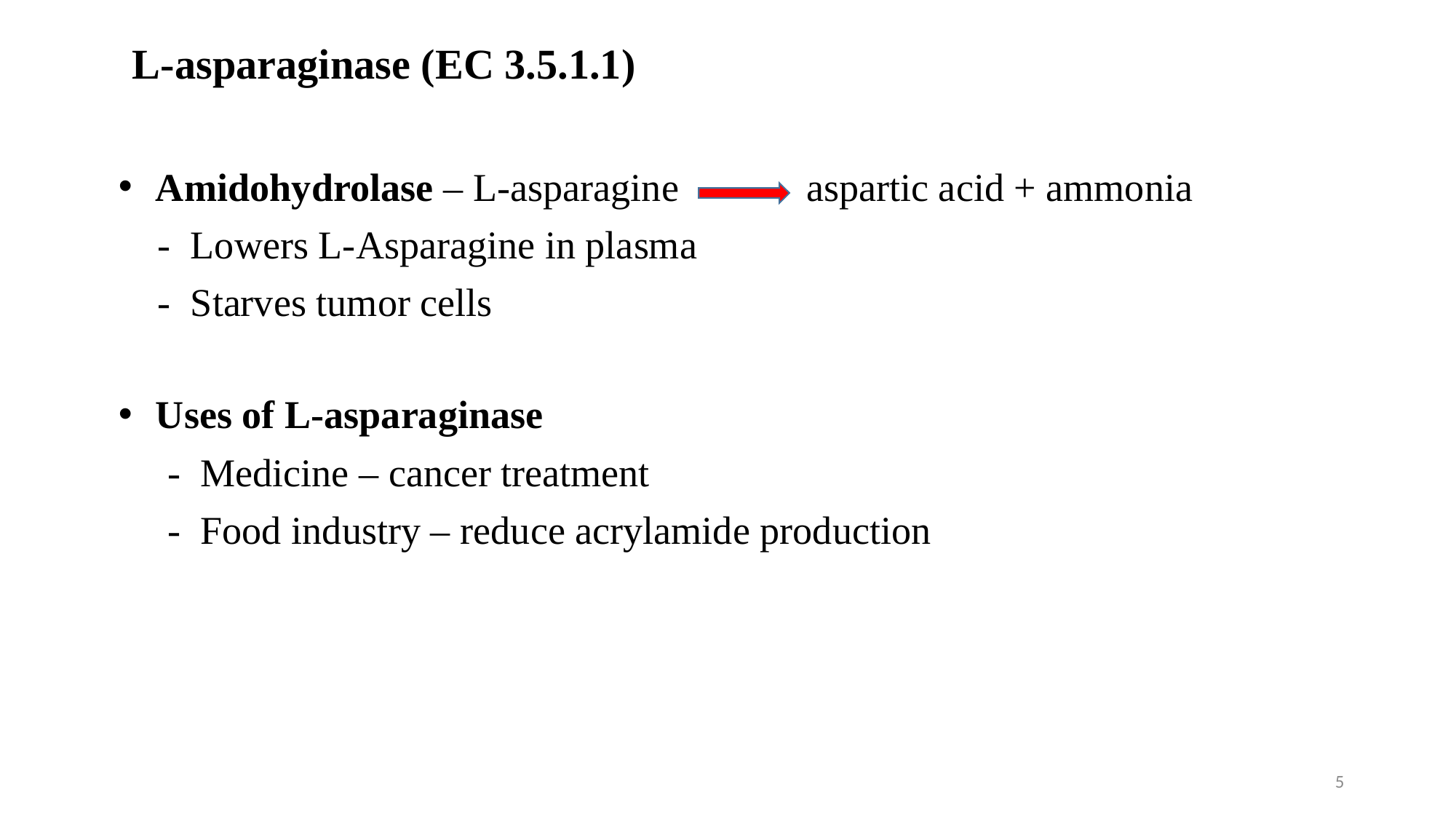

# L-asparaginase (EC 3.5.1.1)
 Amidohydrolase – L-asparagine aspartic acid + ammonia
 - Lowers L-Asparagine in plasma
 - Starves tumor cells
 Uses of L-asparaginase
 - Medicine – cancer treatment
 - Food industry – reduce acrylamide production
5

## Slide 6
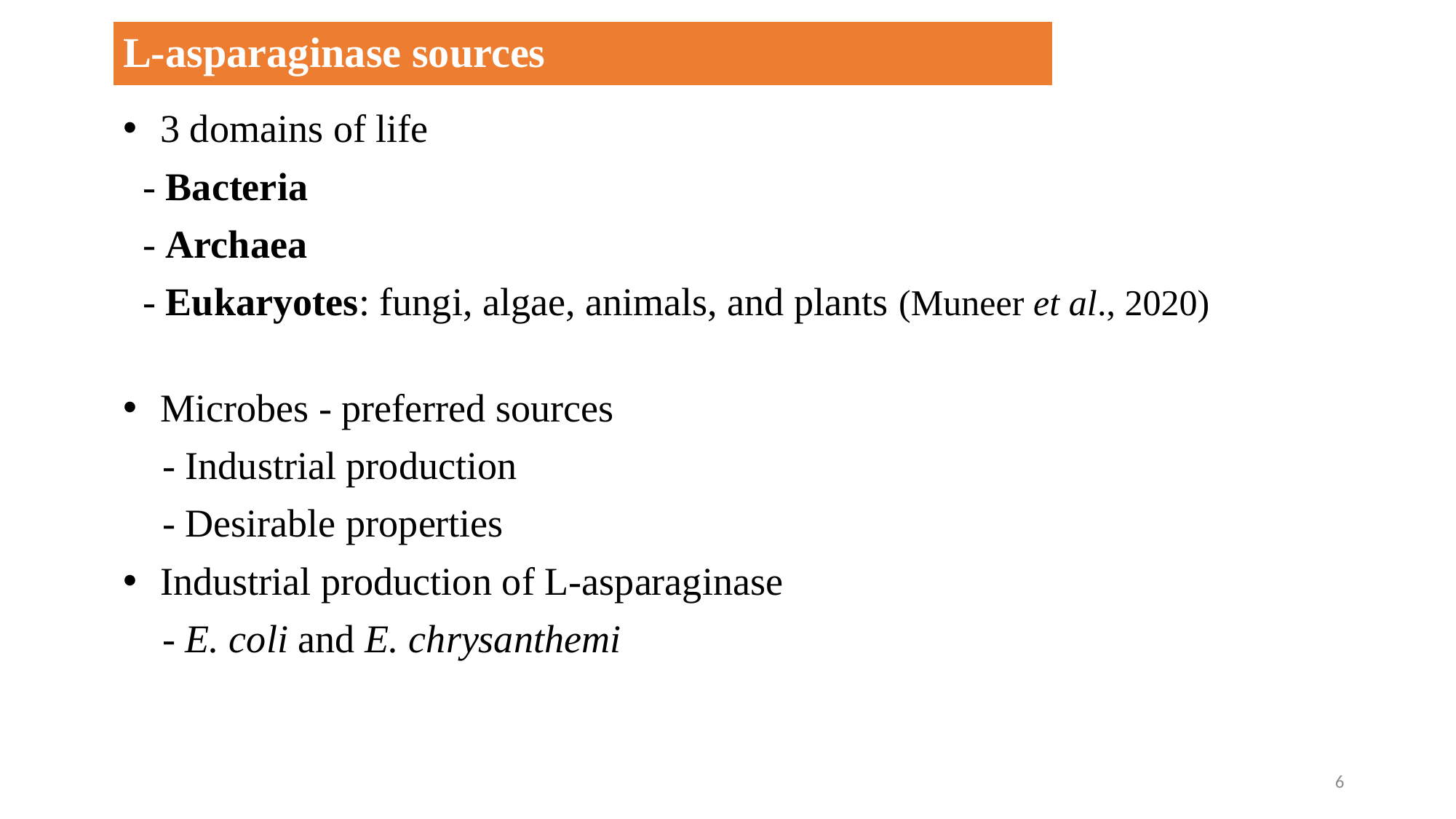

# L-asparaginase sources
 3 domains of life
 - Bacteria
 - Archaea
 - Eukaryotes: fungi, algae, animals, and plants (Muneer et al., 2020)
 Microbes - preferred sources
 - Industrial production
 - Desirable properties
 Industrial production of L-asparaginase
 - E. coli and E. chrysanthemi
6

## Slide 7
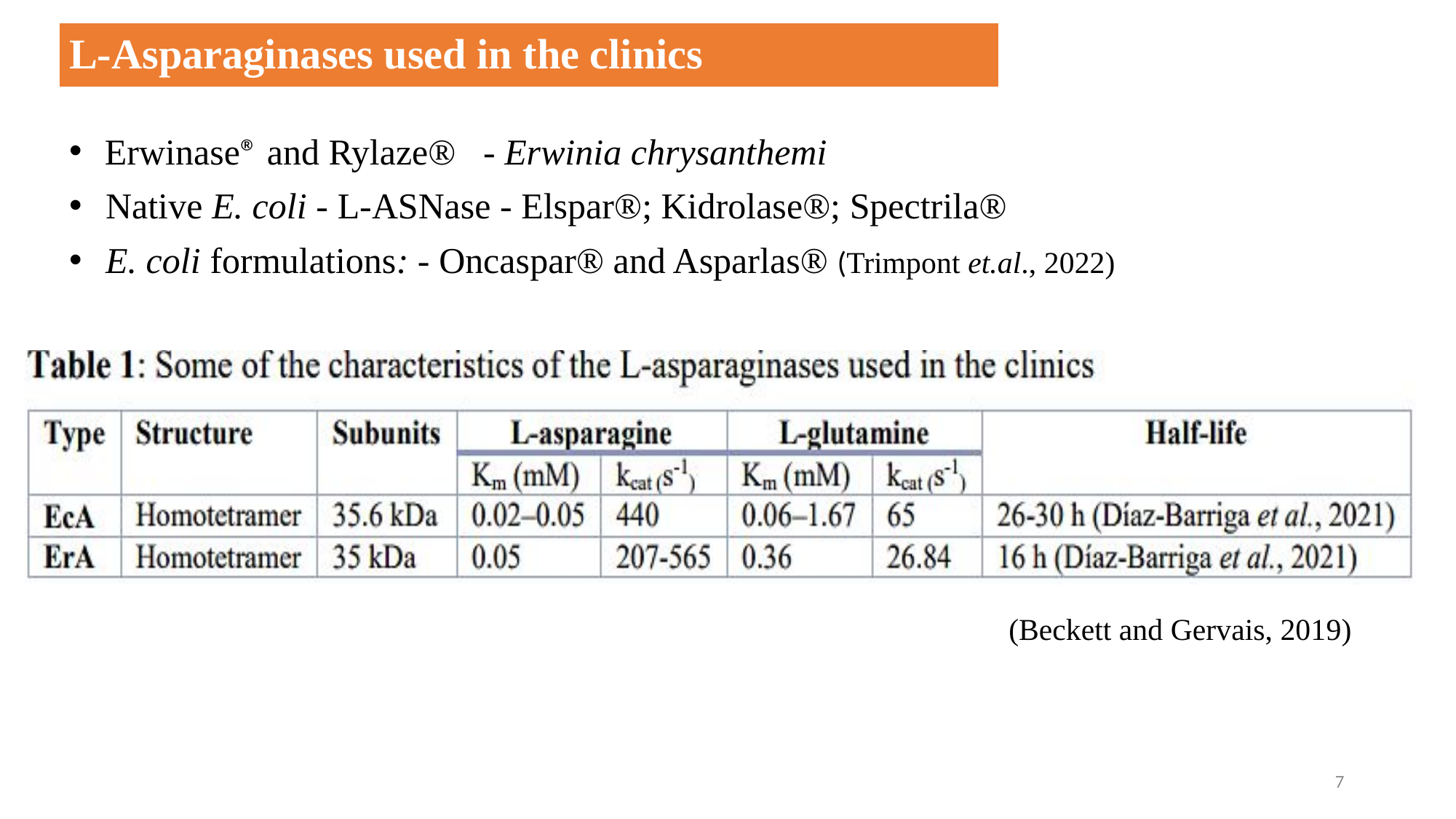

# L-Asparaginases used in the clinics
 Erwinase® and Rylaze® - Erwinia chrysanthemi
 Native E. coli - L-ASNase - Elspar®; Kidrolase®; Spectrila®
 E. coli formulations: - Oncaspar® and Asparlas® (Trimpont et.al., 2022)
(Beckett and Gervais, 2019)
7

## Slide 8
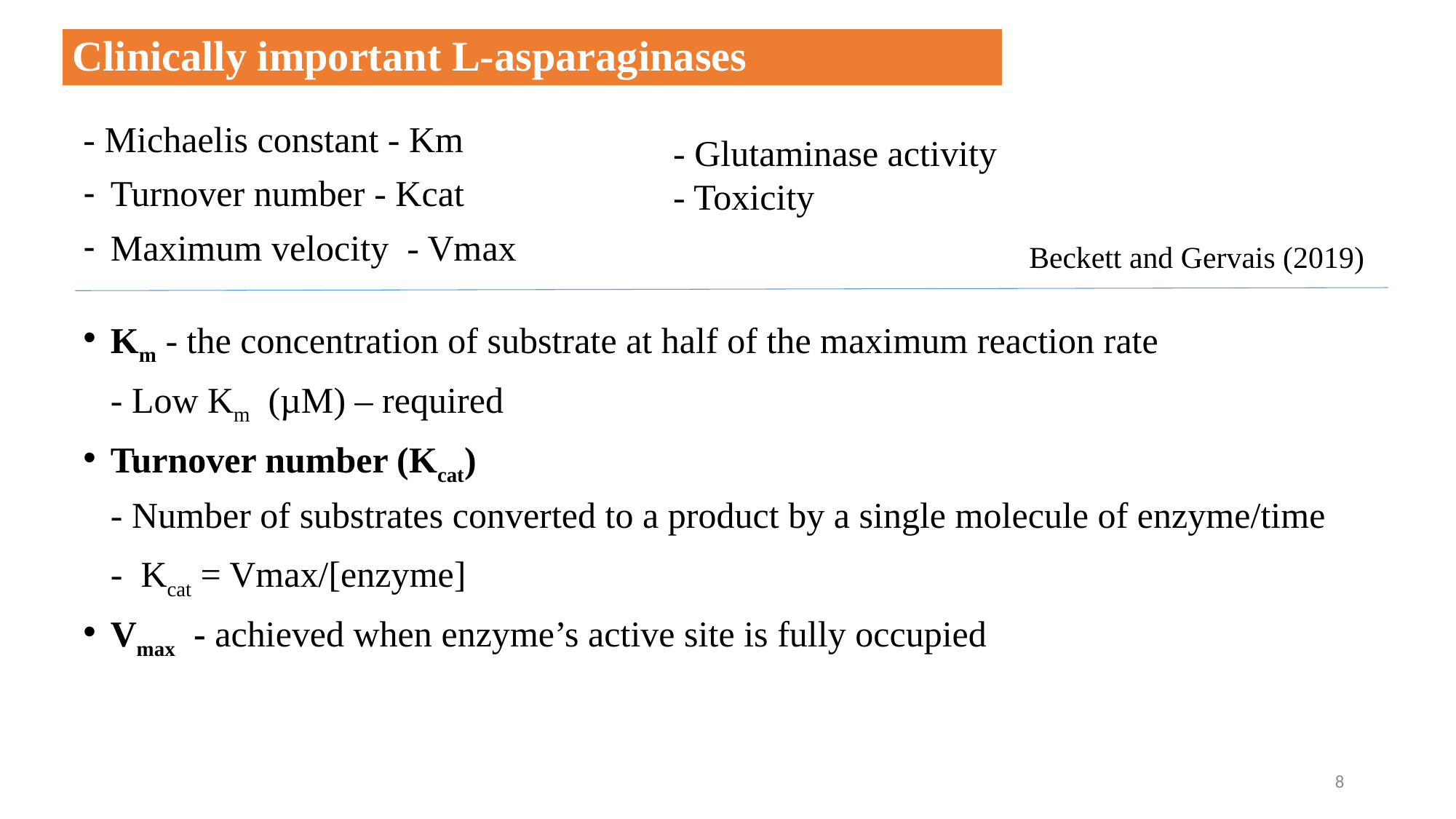

# Clinically important L-asparaginases
- Michaelis constant - Km
Turnover number - Kcat
Maximum velocity - Vmax
Km - the concentration of substrate at half of the maximum reaction rate
 - Low Km (µM) – required
Turnover number (Kcat)
 - Number of substrates converted to a product by a single molecule of enzyme/time
 - Kcat = Vmax/[enzyme]
Vmax - achieved when enzyme’s active site is fully occupied
- Glutaminase activity
- Toxicity
Beckett and Gervais (2019)
8

## Slide 9
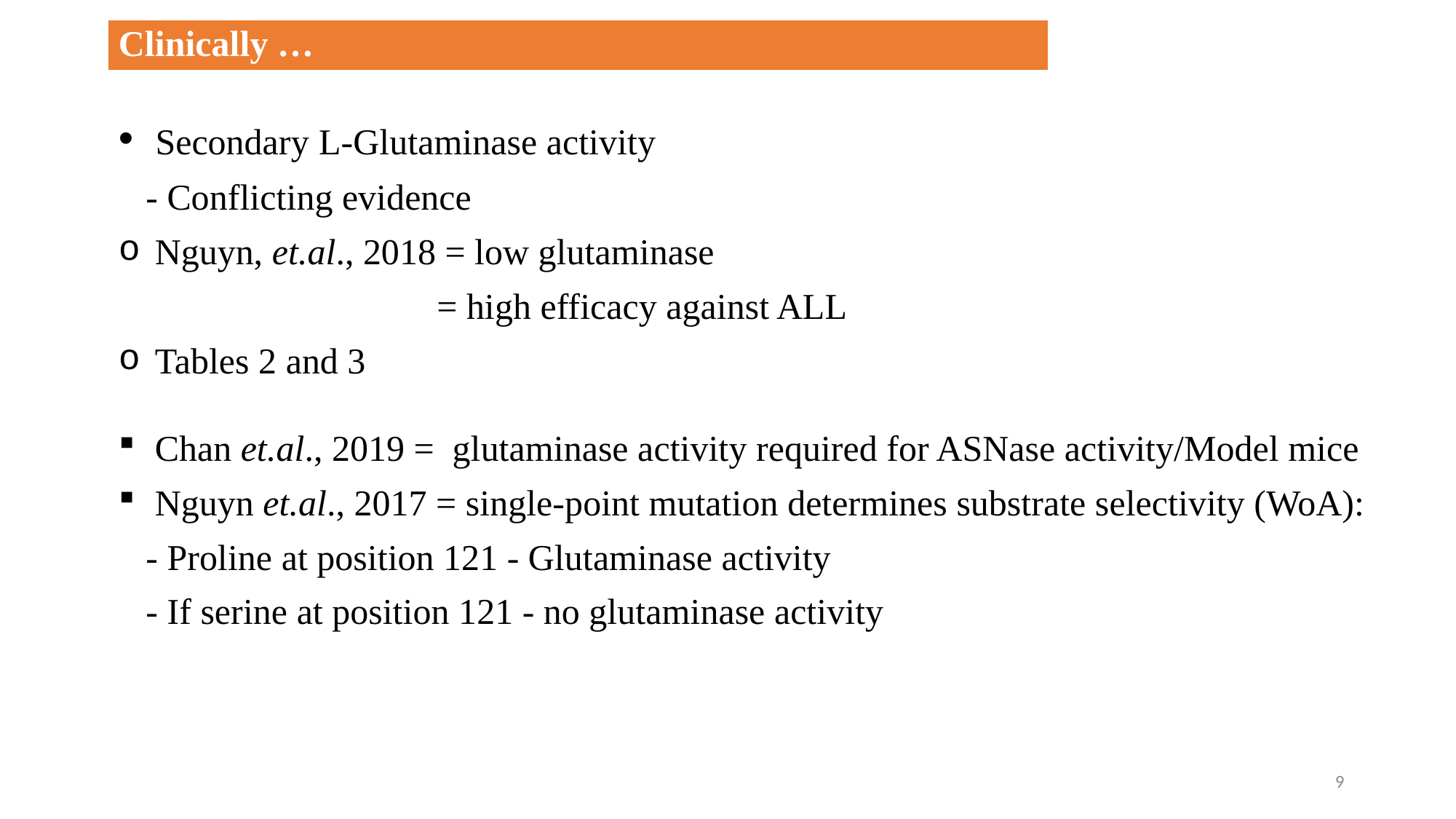

# Clinically …
 Secondary L-Glutaminase activity
 - Conflicting evidence
 Nguyn, et.al., 2018 = low glutaminase
 = high efficacy against ALL
 Tables 2 and 3
 Chan et.al., 2019 = glutaminase activity required for ASNase activity/Model mice
 Nguyn et.al., 2017 = single-point mutation determines substrate selectivity (WoA):
 - Proline at position 121 - Glutaminase activity
 - If serine at position 121 - no glutaminase activity
9

## Slide 10
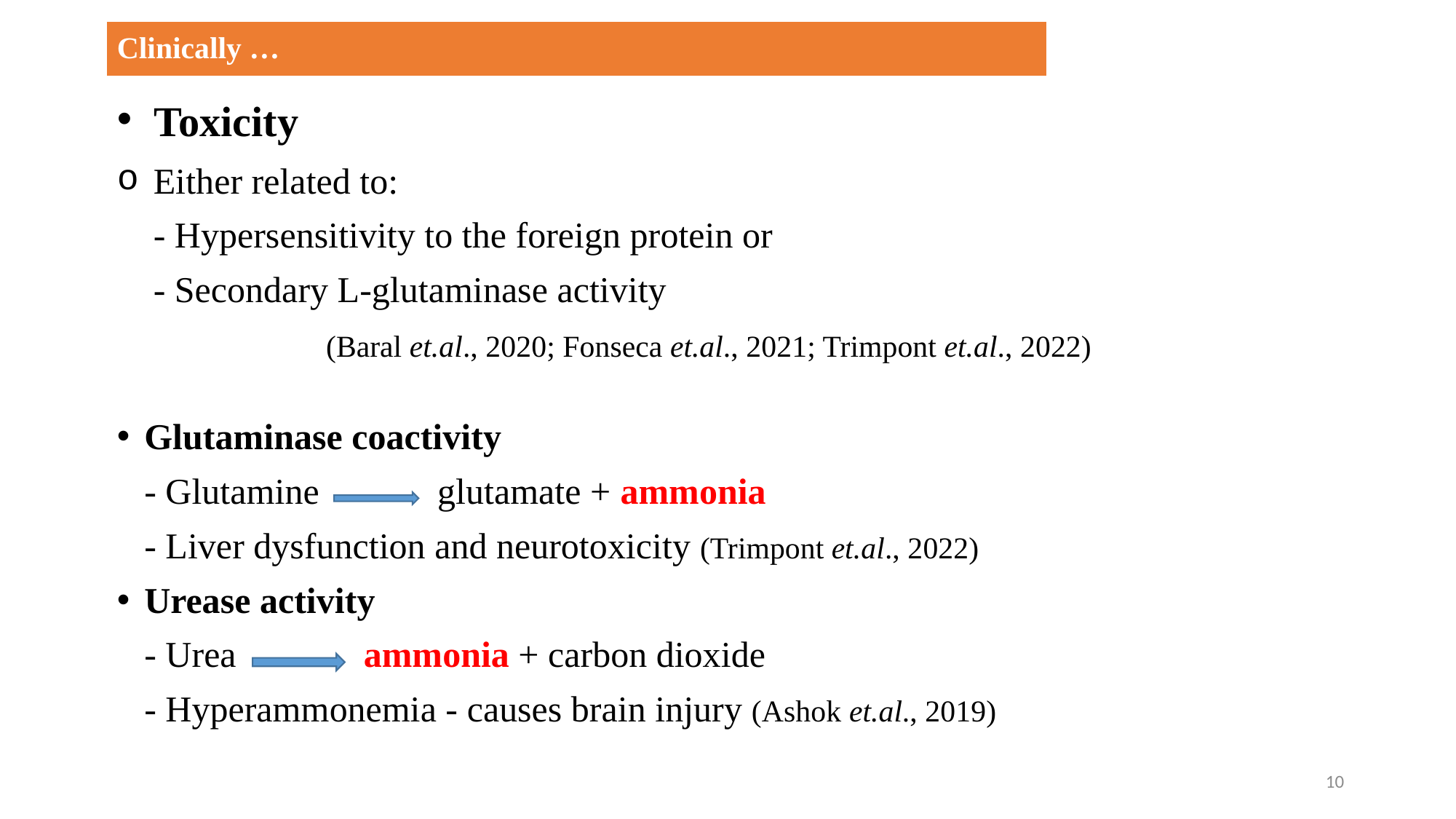

# Clinically …
 Toxicity
 Either related to:
 - Hypersensitivity to the foreign protein or
 - Secondary L-glutaminase activity
 (Baral et.al., 2020; Fonseca et.al., 2021; Trimpont et.al., 2022)
Glutaminase coactivity
 - Glutamine glutamate + ammonia
 - Liver dysfunction and neurotoxicity (Trimpont et.al., 2022)
Urease activity
 - Urea ammonia + carbon dioxide
 - Hyperammonemia - causes brain injury (Ashok et.al., 2019)
10

## Slide 11
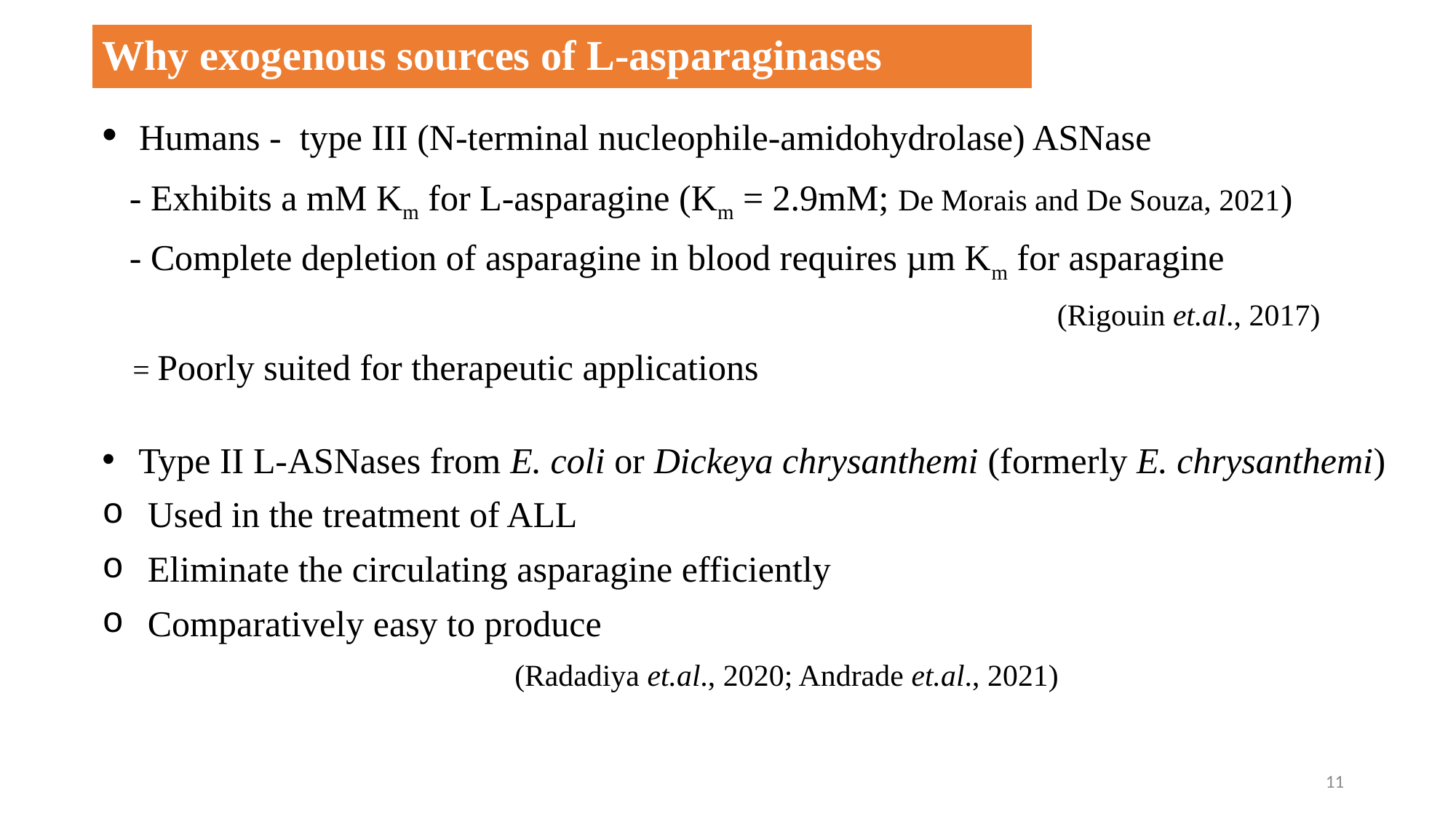

# Why exogenous sources of L-asparaginases
 Humans - type III (N-terminal nucleophile-amidohydrolase) ASNase
 - Exhibits a mM Km for L-asparagine (Km = 2.9mM; De Morais and De Souza, 2021)
 - Complete depletion of asparagine in blood requires µm Km for asparagine
 (Rigouin et.al., 2017)
 = Poorly suited for therapeutic applications
 Type II L-ASNases from E. coli or Dickeya chrysanthemi (formerly E. chrysanthemi)
 Used in the treatment of ALL
 Eliminate the circulating asparagine efficiently
 Comparatively easy to produce
 (Radadiya et.al., 2020; Andrade et.al., 2021)
11

## Slide 12
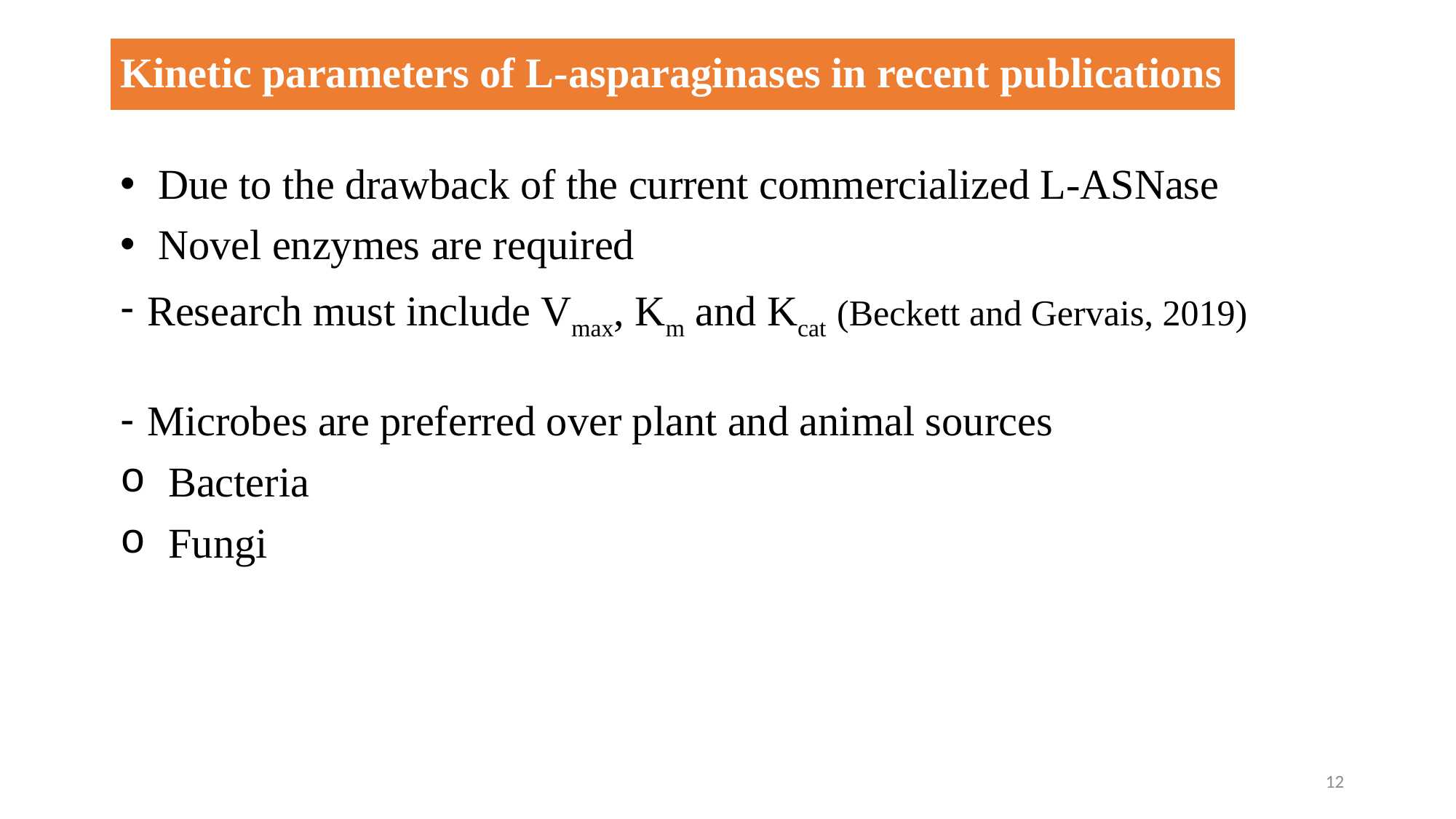

# Kinetic parameters of L-asparaginases in recent publications
 Due to the drawback of the current commercialized L-ASNase
 Novel enzymes are required
Research must include Vmax, Km and Kcat (Beckett and Gervais, 2019)
Microbes are preferred over plant and animal sources
 Bacteria
 Fungi
12

## Slide 13
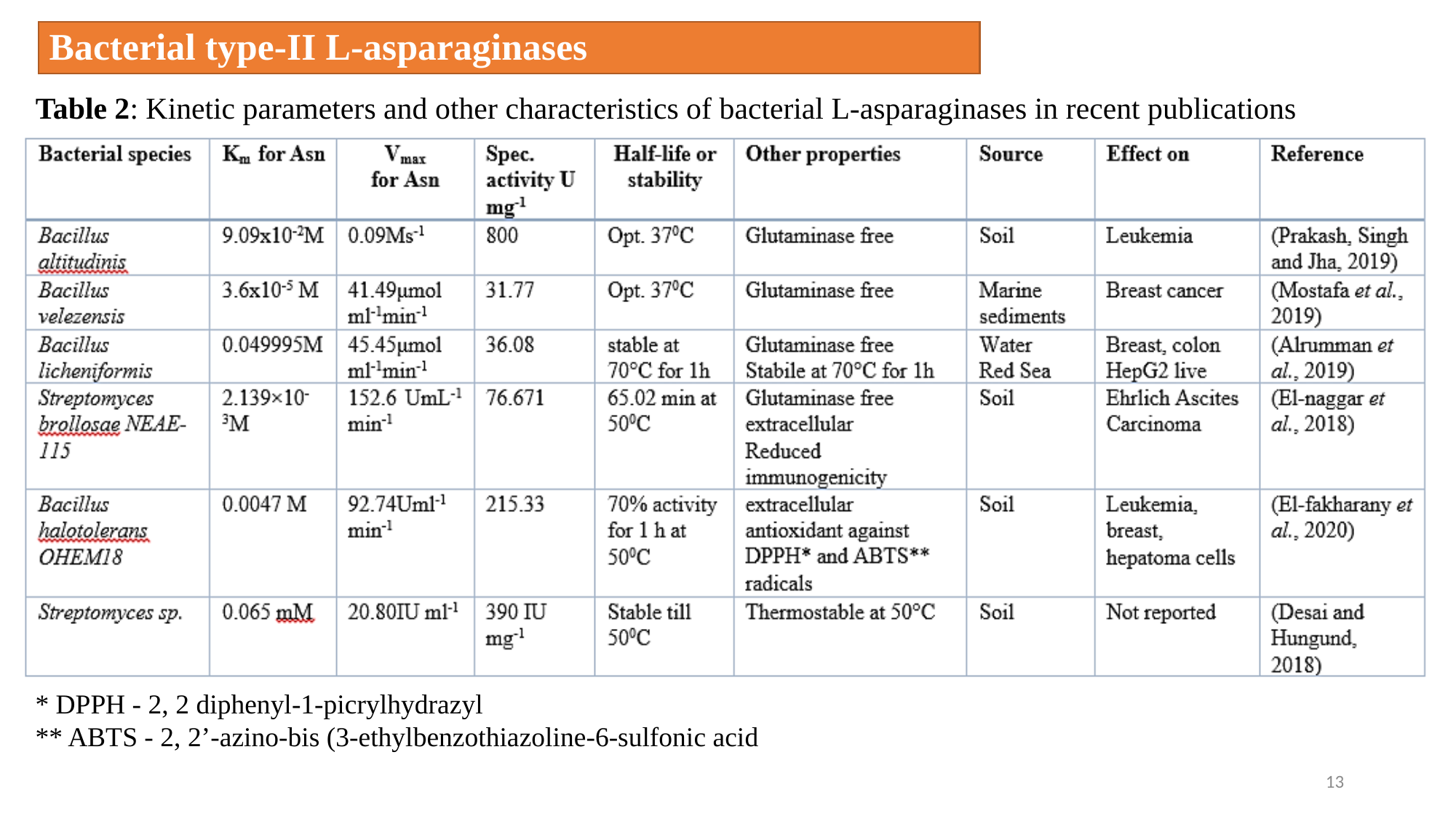

# Bacterial type-II L-asparaginases
Table 2: Kinetic parameters and other characteristics of bacterial L-asparaginases in recent publications
* DPPH - 2, 2 diphenyl-1-picrylhydrazyl
** ABTS - 2, 2’-azino-bis (3-ethylbenzothiazoline-6-sulfonic acid
13

## Slide 14
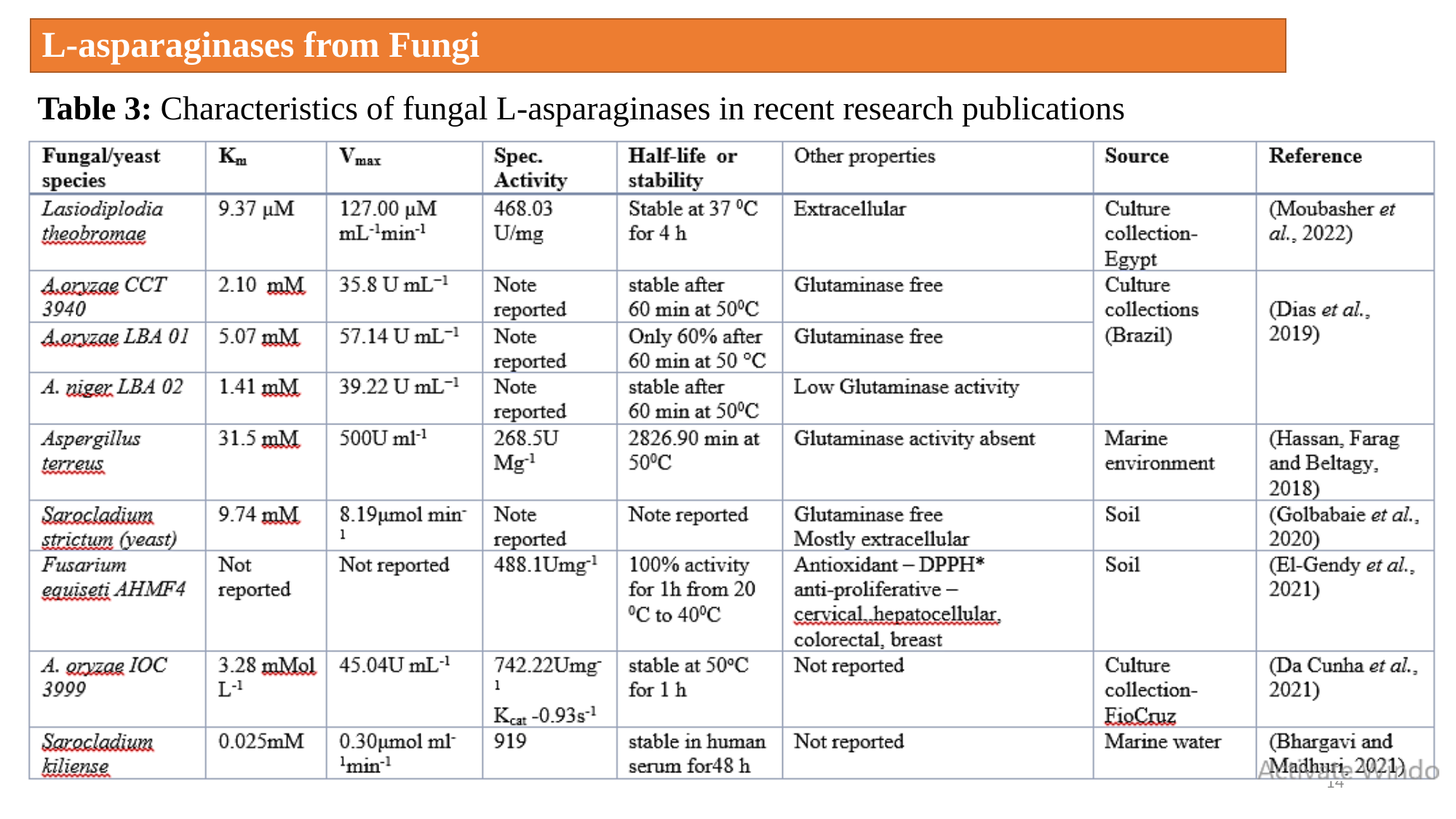

# L-asparaginases from Fungi
Table 3: Characteristics of fungal L-asparaginases in recent research publications
14

## Slide 15
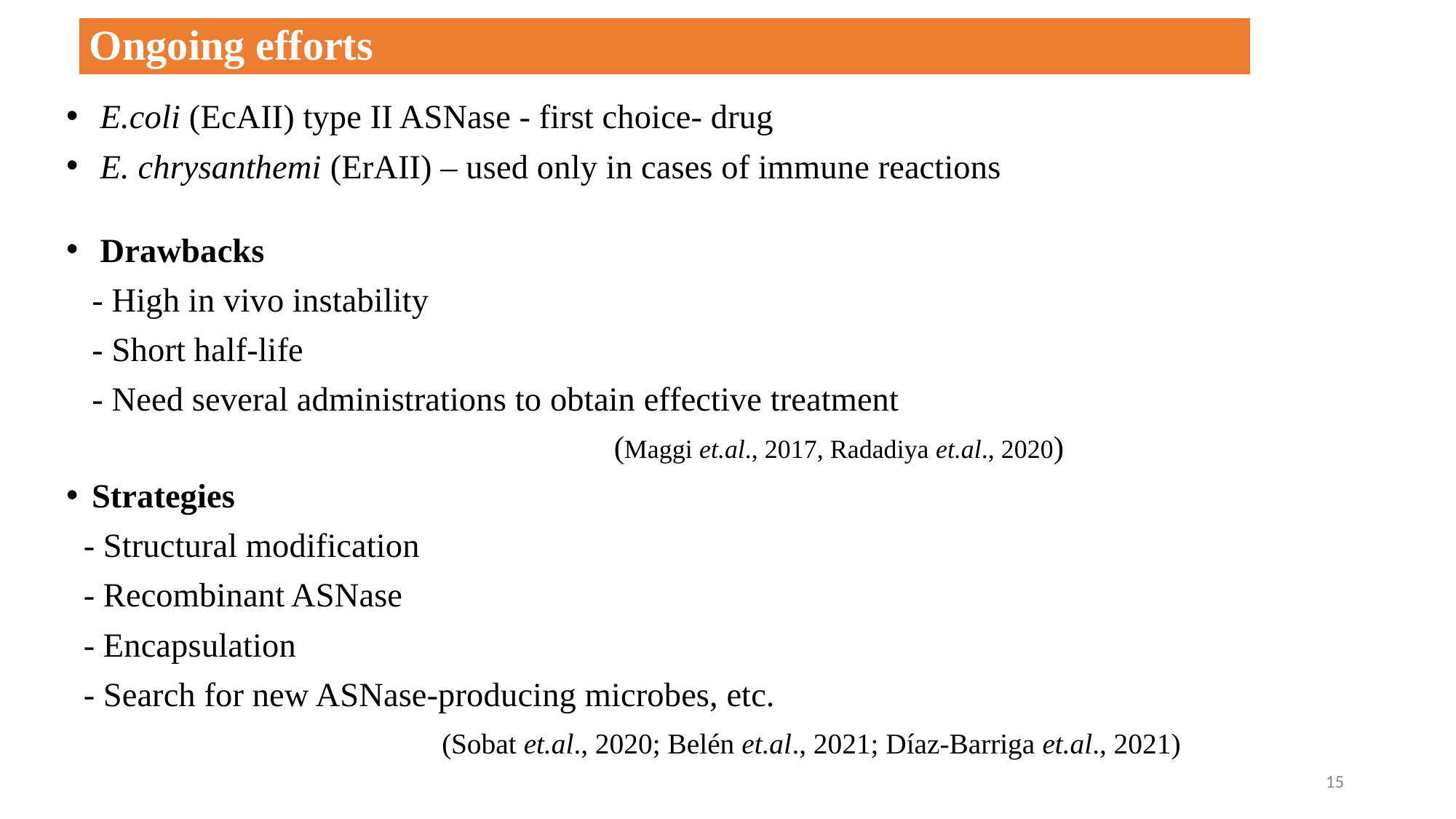

# Ongoing efforts
 E.coli (EcAII) type II ASNase - first choice- drug
 E. chrysanthemi (ErAII) – used only in cases of immune reactions
 Drawbacks
 - High in vivo instability
 - Short half-life
 - Need several administrations to obtain effective treatment
 (Maggi et.al., 2017, Radadiya et.al., 2020)
Strategies
 - Structural modification
 - Recombinant ASNase
 - Encapsulation
 - Search for new ASNase-producing microbes, etc.
 (Sobat et.al., 2020; Belén et.al., 2021; Díaz-Barriga et.al., 2021)
15

## Slide 16
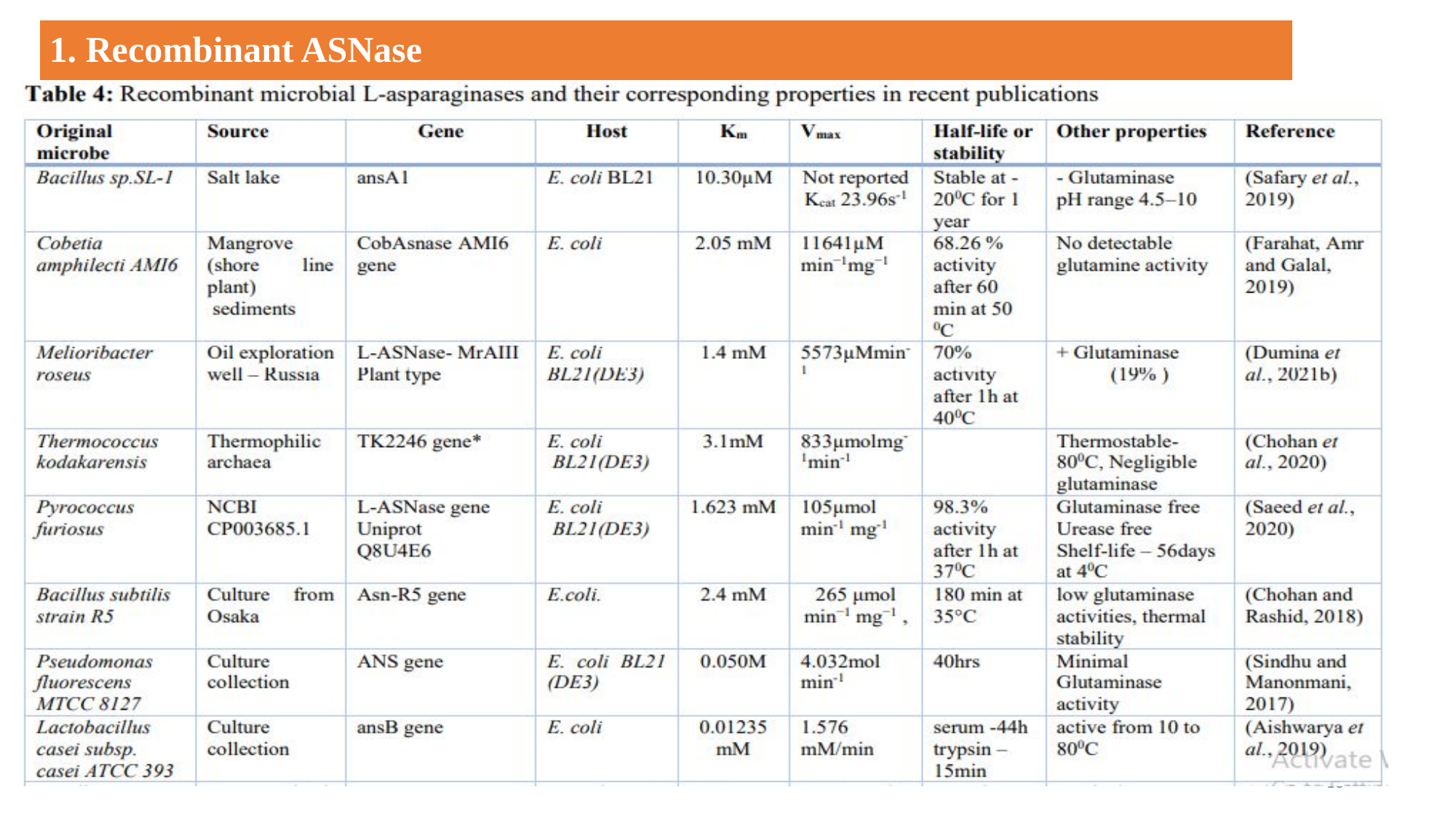

# 1. Recombinant ASNase
16

## Slide 17
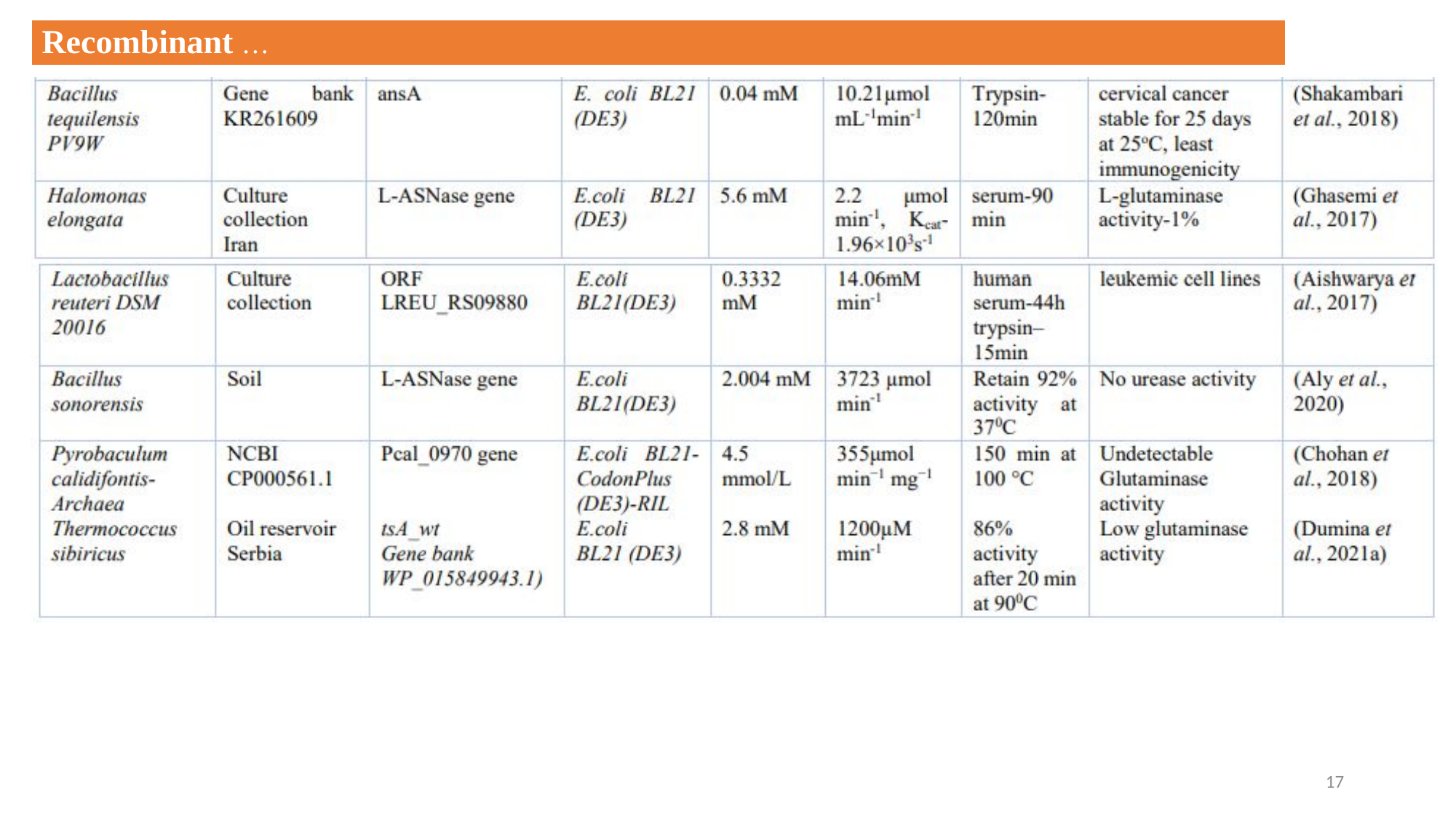

# Recombinant …
17

## Slide 18
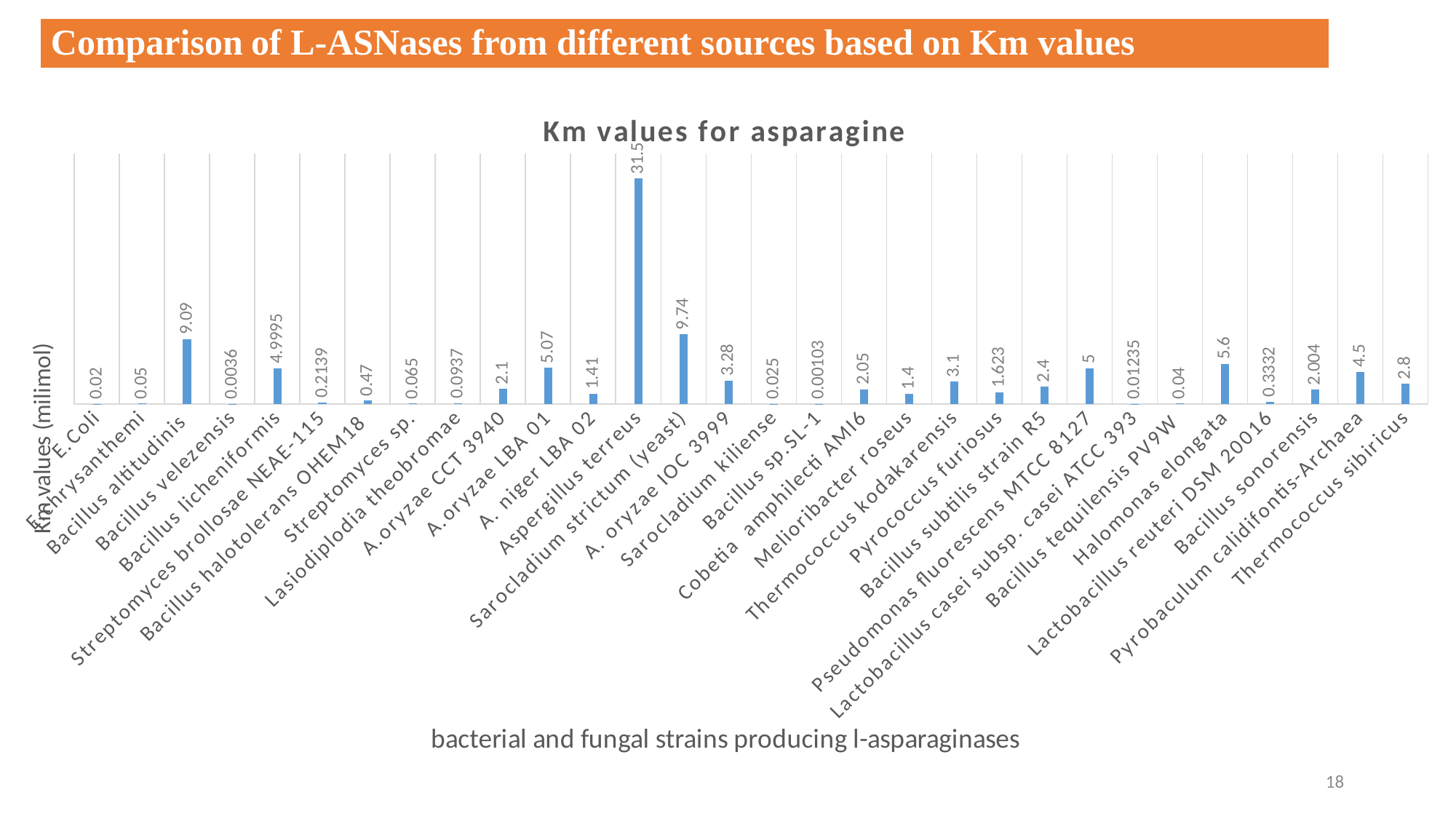

# Comparison of L-ASNases from different sources based on Km values
### Chart: Km values for asparagine
| Category | |
|---|---|
| E.Coli | 0.02 |
| E.chrysanthemi | 0.05 |
| Bacillus altitudinis | 9.09 |
| Bacillus velezensis | 0.0036 |
| Bacillus licheniformis | 4.9995 |
| Streptomyces brollosae NEAE-115 | 0.2139 |
| Bacillus halotolerans OHEM18 | 0.47 |
| Streptomyces sp. | 0.065 |
| Lasiodiplodia theobromae | 0.0937 |
| A.oryzae CCT 3940 | 2.1 |
| A.oryzae LBA 01 | 5.07 |
| A. niger LBA 02 | 1.41 |
| Aspergillus terreus | 31.5 |
| Sarocladium strictum (yeast) | 9.74 |
| A. oryzae IOC 3999 | 3.28 |
| Sarocladium kiliense | 0.025 |
| Bacillus sp.SL-1 | 0.00103 |
| Cobetia amphilecti AMI6 | 2.05 |
| Melioribacter roseus | 1.4 |
| Thermococcus kodakarensis | 3.1 |
| Pyrococcus furiosus | 1.623 |
| Bacillus subtilis strain R5 | 2.4 |
| Pseudomonas fluorescens MTCC 8127 | 5.0 |
| Lactobacillus casei subsp. casei ATCC 393 | 0.01235 |
| Bacillus tequilensis PV9W | 0.04 |
| Halomonas elongata | 5.6 |
| Lactobacillus reuteri DSM 20016 | 0.3332 |
| Bacillus sonorensis | 2.004 |
| Pyrobaculum calidifontis-Archaea | 4.5 |
| Thermococcus sibiricus | 2.8 |18

## Slide 19
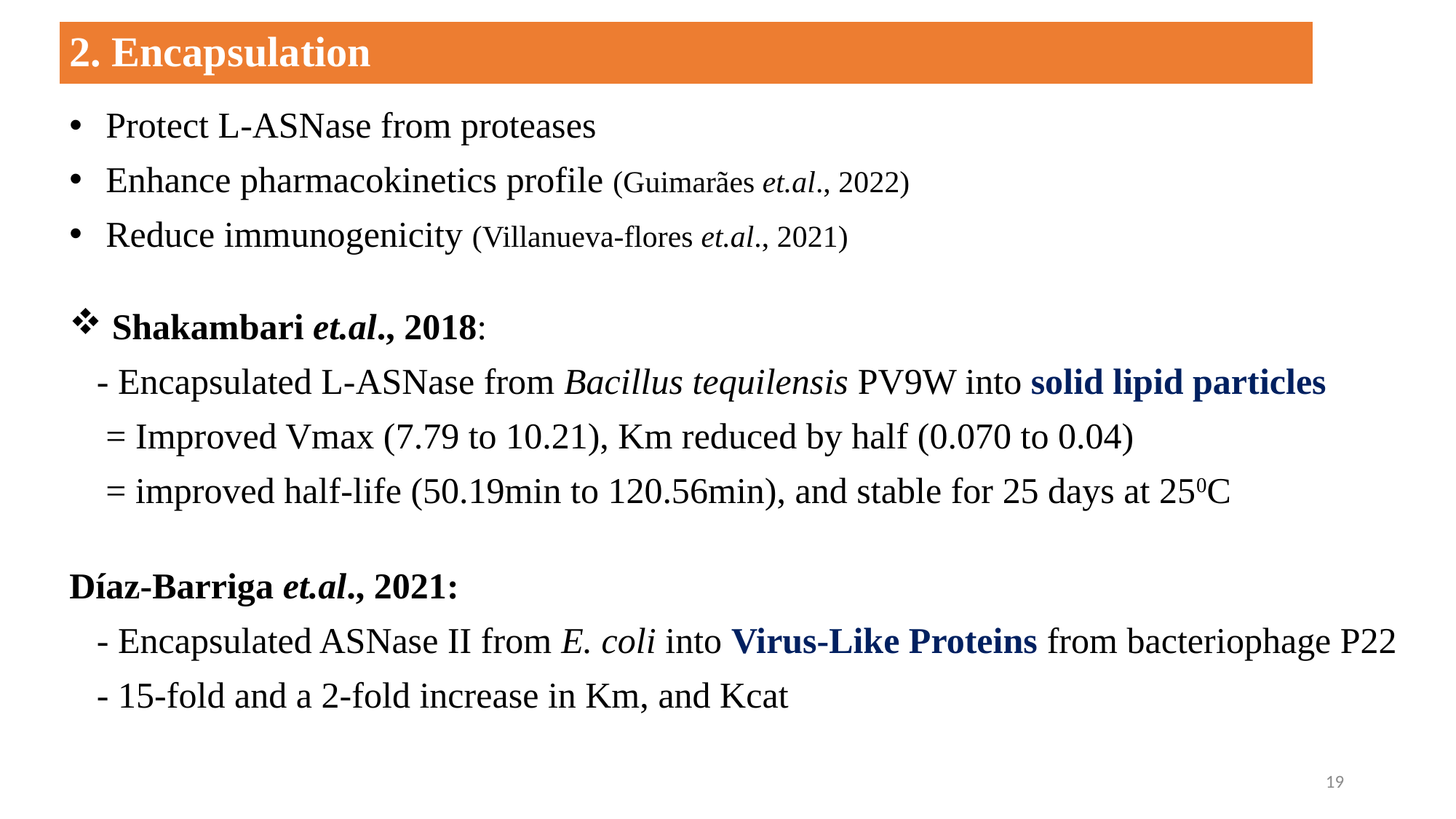

# 2. Encapsulation
 Protect L-ASNase from proteases
 Enhance pharmacokinetics profile (Guimarães et.al., 2022)
 Reduce immunogenicity (Villanueva-flores et.al., 2021)
 Shakambari et.al., 2018:
 - Encapsulated L-ASNase from Bacillus tequilensis PV9W into solid lipid particles
 = Improved Vmax (7.79 to 10.21), Km reduced by half (0.070 to 0.04)
 = improved half-life (50.19min to 120.56min), and stable for 25 days at 250C
Díaz-Barriga et.al., 2021:
 - Encapsulated ASNase II from E. coli into Virus-Like Proteins from bacteriophage P22
 - 15-fold and a 2-fold increase in Km, and Kcat
19

## Slide 20
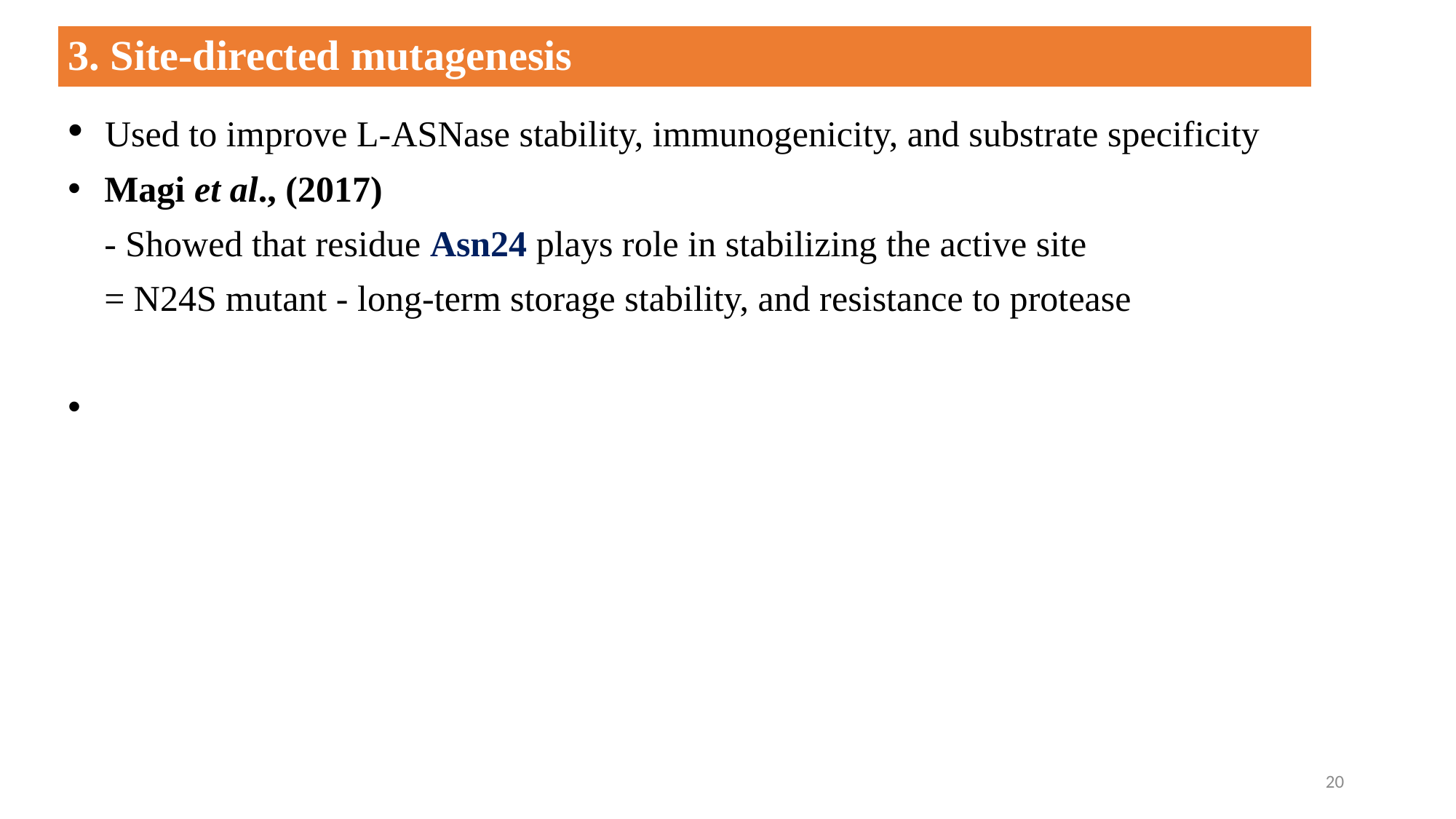

# 3. Site-directed mutagenesis
 Used to improve L-ASNase stability, immunogenicity, and substrate specificity
 Magi et al., (2017)
 - Showed that residue Asn24 plays role in stabilizing the active site
 = N24S mutant - long-term storage stability, and resistance to protease
20

## Slide 21
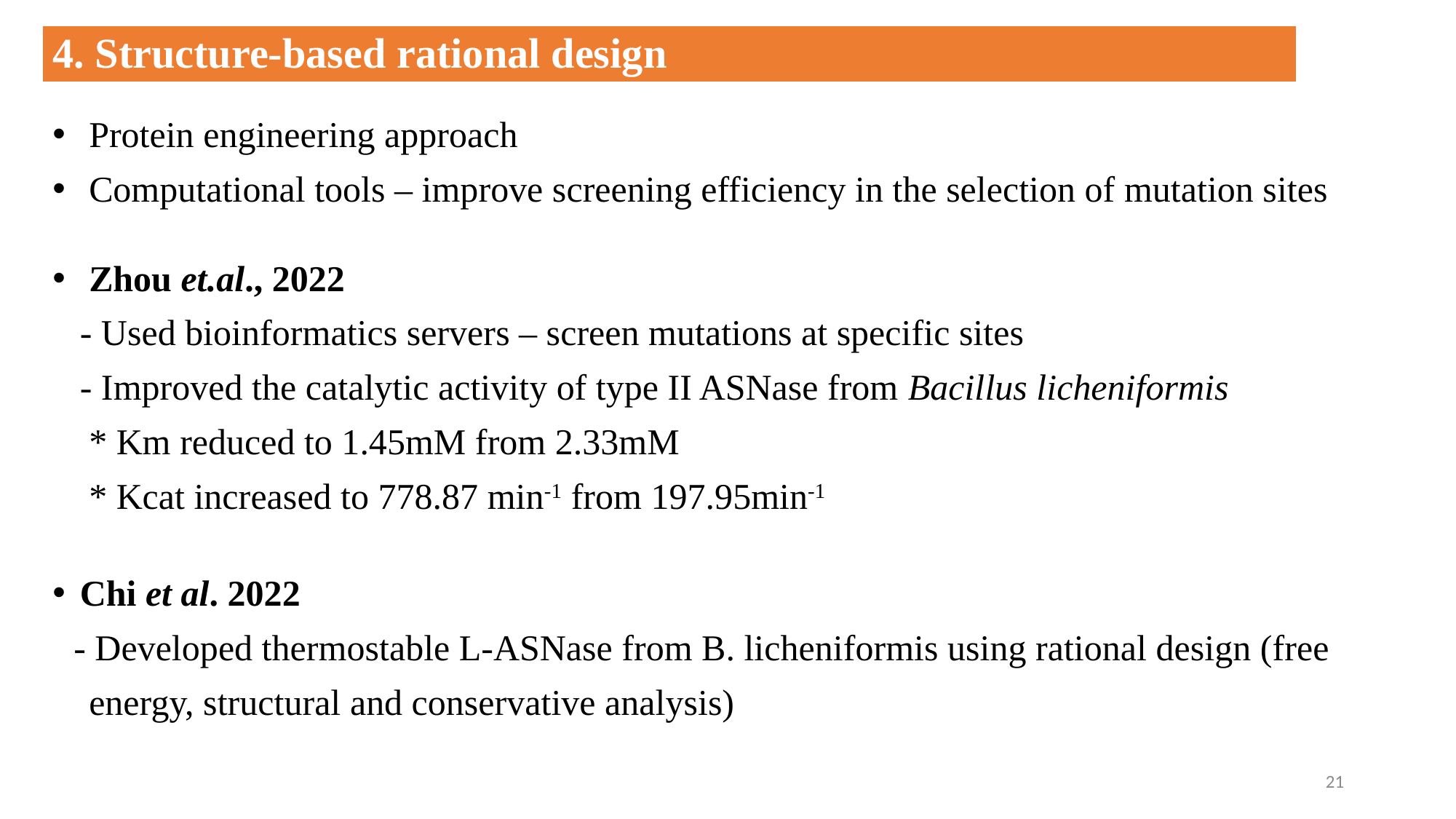

# 4. Structure-based rational design
 Protein engineering approach
 Computational tools – improve screening efficiency in the selection of mutation sites
 Zhou et.al., 2022
 - Used bioinformatics servers – screen mutations at specific sites
 - Improved the catalytic activity of type II ASNase from Bacillus licheniformis
 * Km reduced to 1.45mM from 2.33mM
 * Kcat increased to 778.87 min-1 from 197.95min-1
Chi et al. 2022
 - Developed thermostable L-ASNase from B. licheniformis using rational design (free
 energy, structural and conservative analysis)
21

## Slide 22
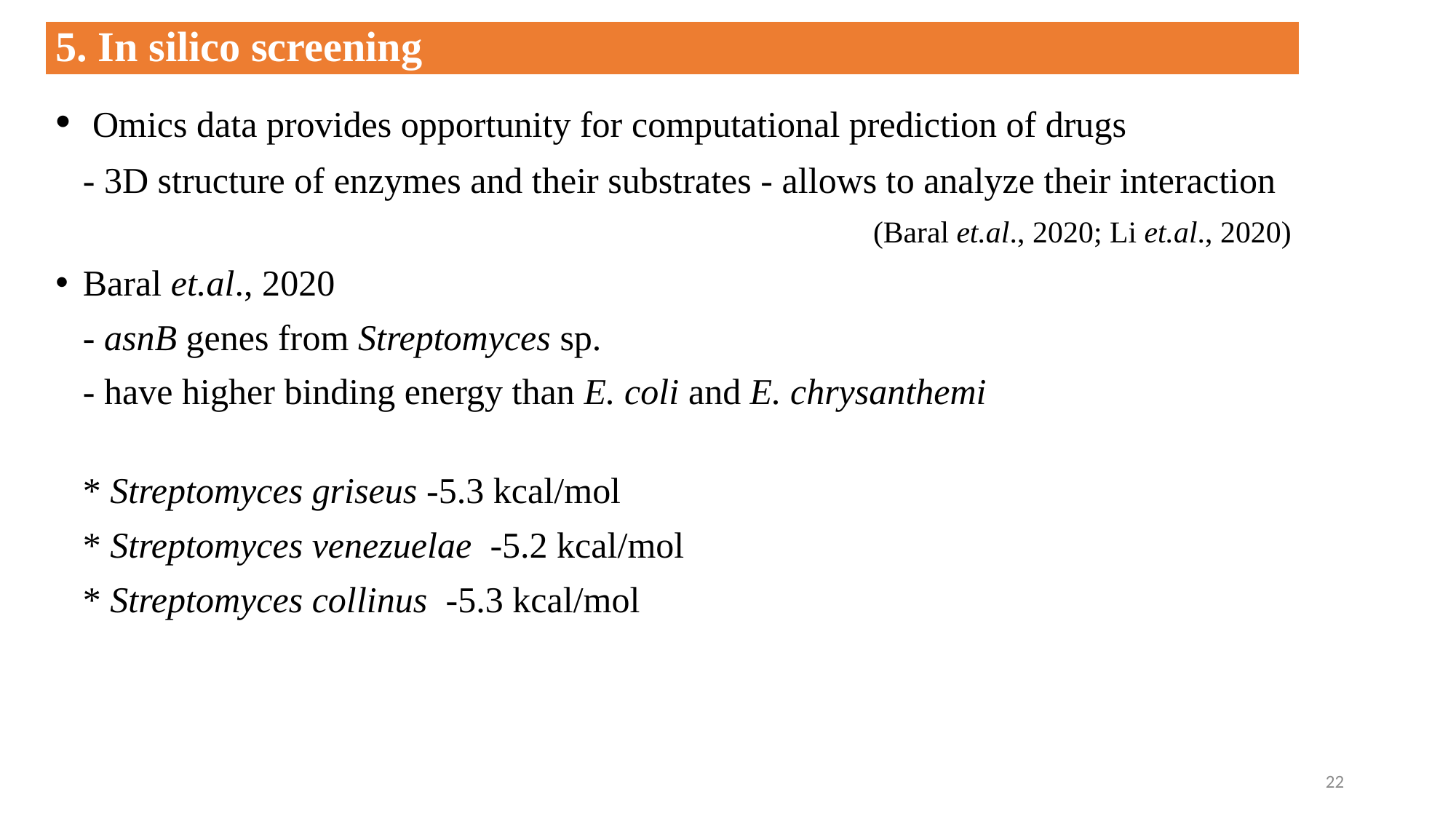

# 5. In silico screening
 Omics data provides opportunity for computational prediction of drugs
 - 3D structure of enzymes and their substrates - allows to analyze their interaction
 (Baral et.al., 2020; Li et.al., 2020)
Baral et.al., 2020
 - asnB genes from Streptomyces sp.
 - have higher binding energy than E. coli and E. chrysanthemi
 * Streptomyces griseus -5.3 kcal/mol
 * Streptomyces venezuelae -5.2 kcal/mol
 * Streptomyces collinus -5.3 kcal/mol
22

## Slide 23
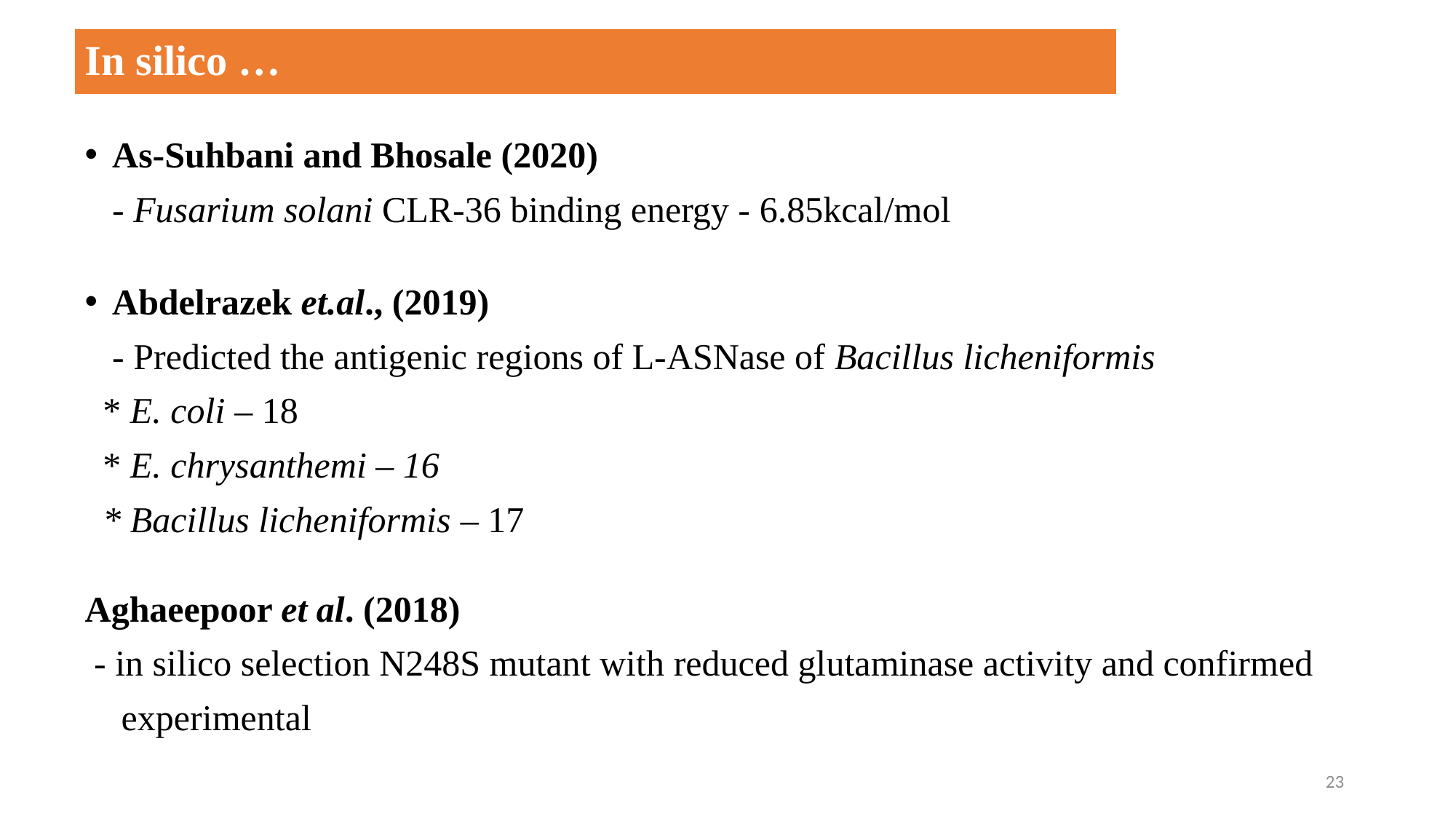

# In silico …
As-Suhbani and Bhosale (2020)
 - Fusarium solani CLR-36 binding energy - 6.85kcal/mol
Abdelrazek et.al., (2019)
 - Predicted the antigenic regions of L-ASNase of Bacillus licheniformis
 * E. coli – 18
 * E. chrysanthemi – 16
 * Bacillus licheniformis – 17
Aghaeepoor et al. (2018)
 - in silico selection N248S mutant with reduced glutaminase activity and confirmed
 experimental
23

## Slide 24
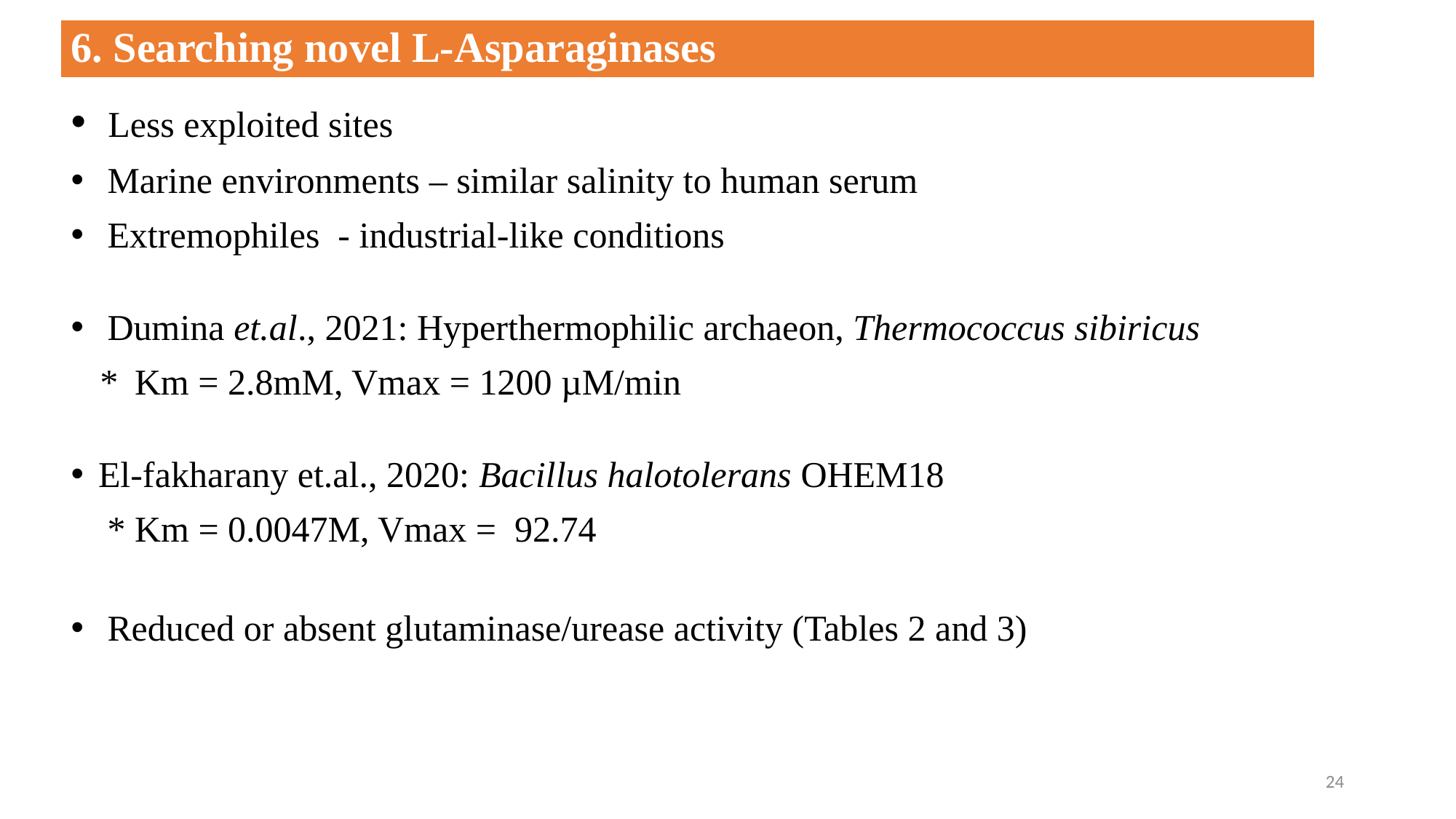

# 6. Searching novel L-Asparaginases
 Less exploited sites
 Marine environments – similar salinity to human serum
 Extremophiles - industrial-like conditions
 Dumina et.al., 2021: Hyperthermophilic archaeon, Thermococcus sibiricus
 * Km = 2.8mM, Vmax = 1200 µM/min
El-fakharany et.al., 2020: Bacillus halotolerans OHEM18
 * Km = 0.0047M, Vmax = 92.74
 Reduced or absent glutaminase/urease activity (Tables 2 and 3)
24

## Slide 25
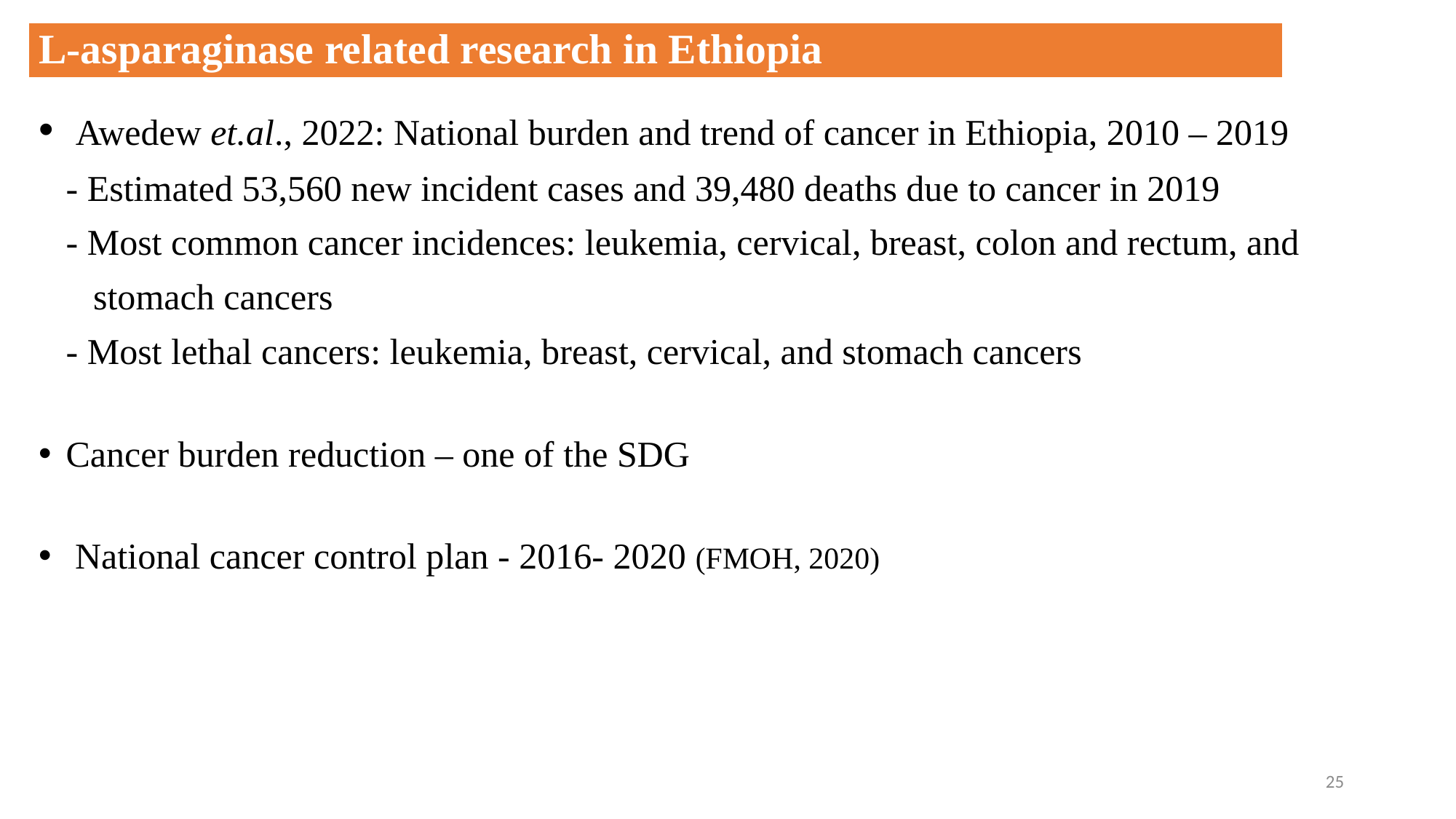

# L-asparaginase related research in Ethiopia
 Awedew et.al., 2022: National burden and trend of cancer in Ethiopia, 2010 – 2019
 - Estimated 53,560 new incident cases and 39,480 deaths due to cancer in 2019
 - Most common cancer incidences: leukemia, cervical, breast, colon and rectum, and
 stomach cancers
 - Most lethal cancers: leukemia, breast, cervical, and stomach cancers
Cancer burden reduction – one of the SDG
 National cancer control plan - 2016- 2020 (FMOH, 2020)
25

## Slide 26
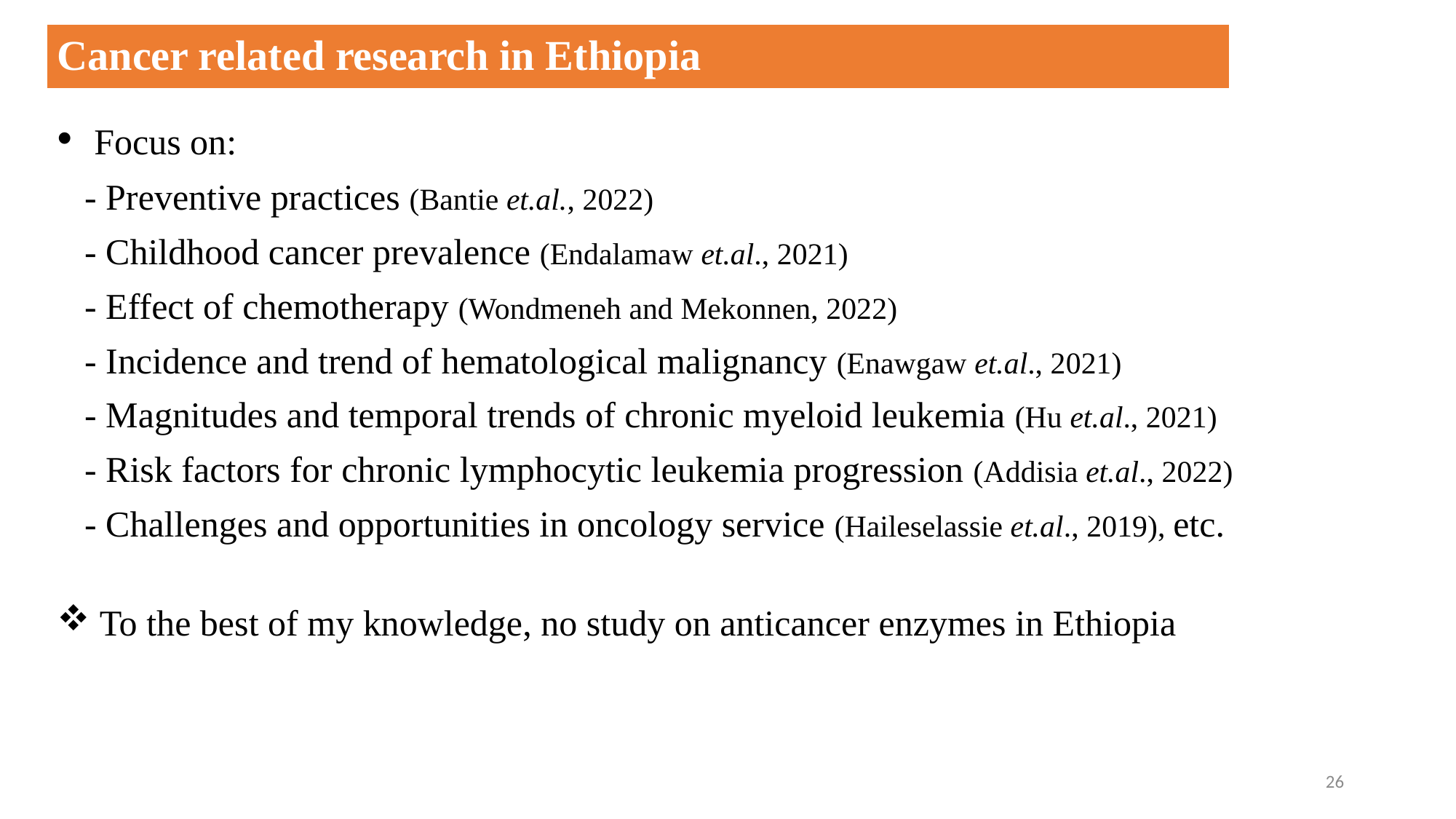

# Cancer related research in Ethiopia
 Focus on:
 - Preventive practices (Bantie et.al., 2022)
 - Childhood cancer prevalence (Endalamaw et.al., 2021)
 - Effect of chemotherapy (Wondmeneh and Mekonnen, 2022)
 - Incidence and trend of hematological malignancy (Enawgaw et.al., 2021)
 - Magnitudes and temporal trends of chronic myeloid leukemia (Hu et.al., 2021)
 - Risk factors for chronic lymphocytic leukemia progression (Addisia et.al., 2022)
 - Challenges and opportunities in oncology service (Haileselassie et.al., 2019), etc.
 To the best of my knowledge, no study on anticancer enzymes in Ethiopia
26

## Slide 27
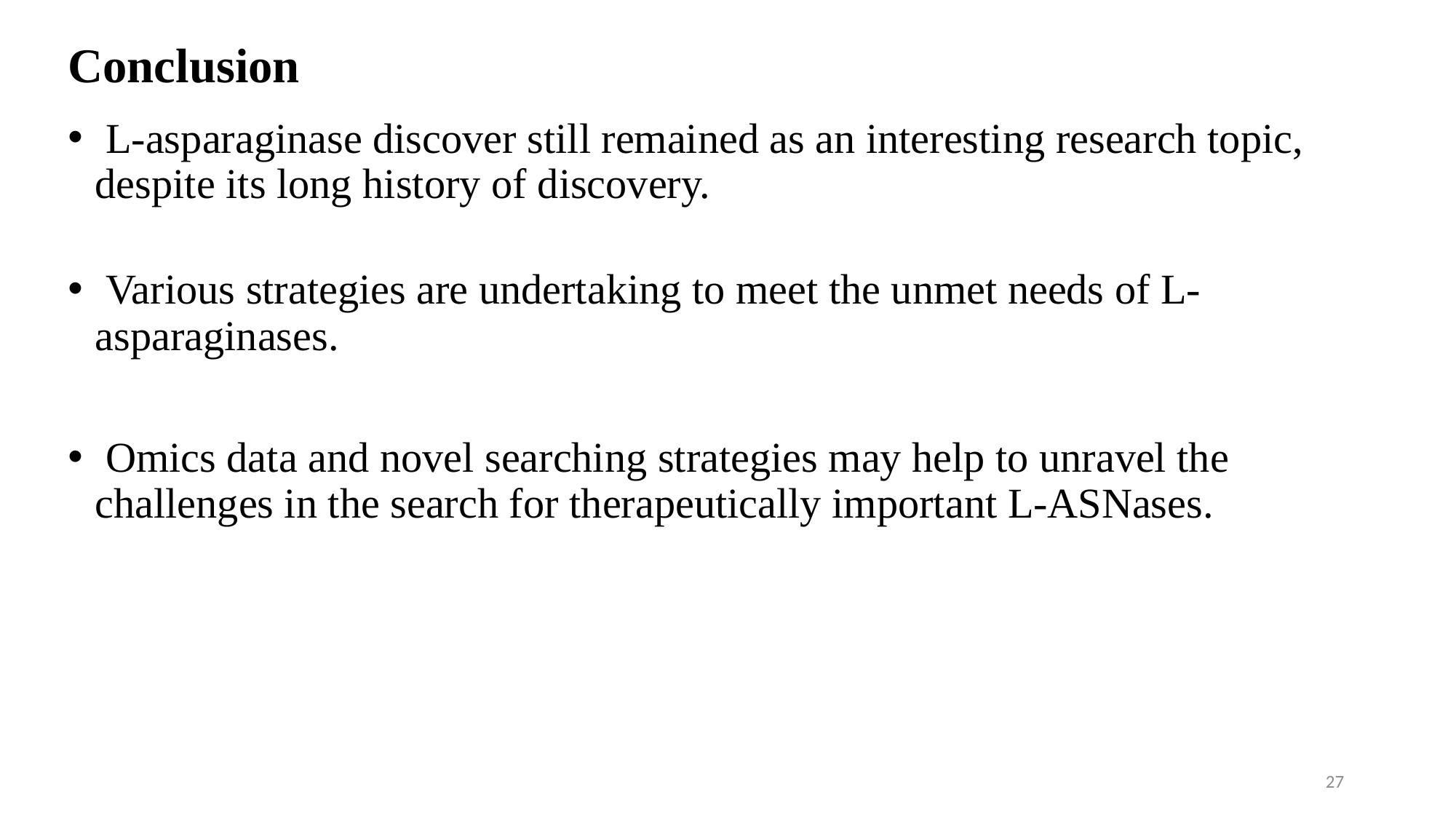

# Conclusion
 L-asparaginase discover still remained as an interesting research topic, despite its long history of discovery.
 Various strategies are undertaking to meet the unmet needs of L-asparaginases.
 Omics data and novel searching strategies may help to unravel the challenges in the search for therapeutically important L-ASNases.
27

## Slide 28
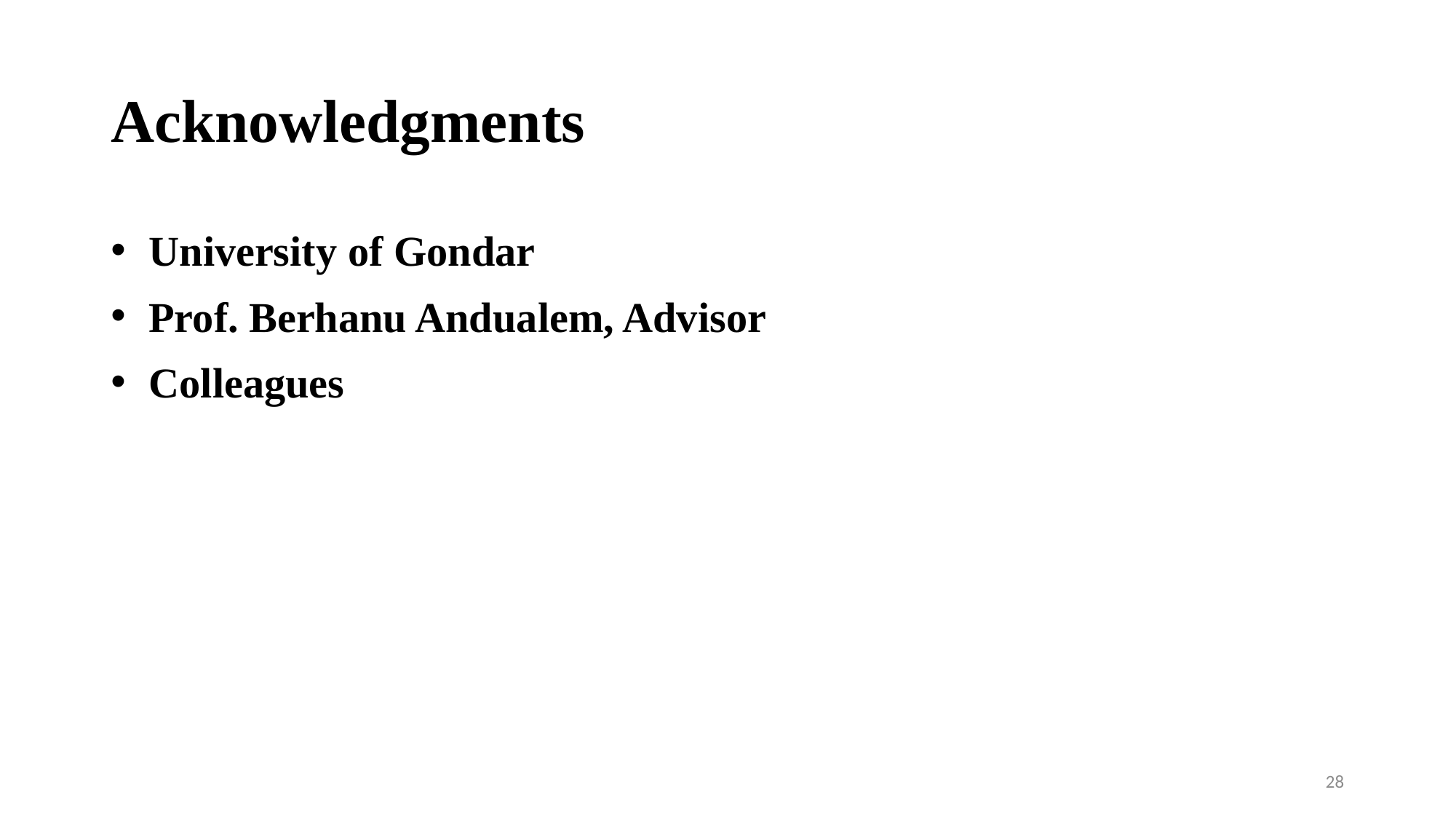

# Acknowledgments
 University of Gondar
 Prof. Berhanu Andualem, Advisor
 Colleagues
28

## Slide 29
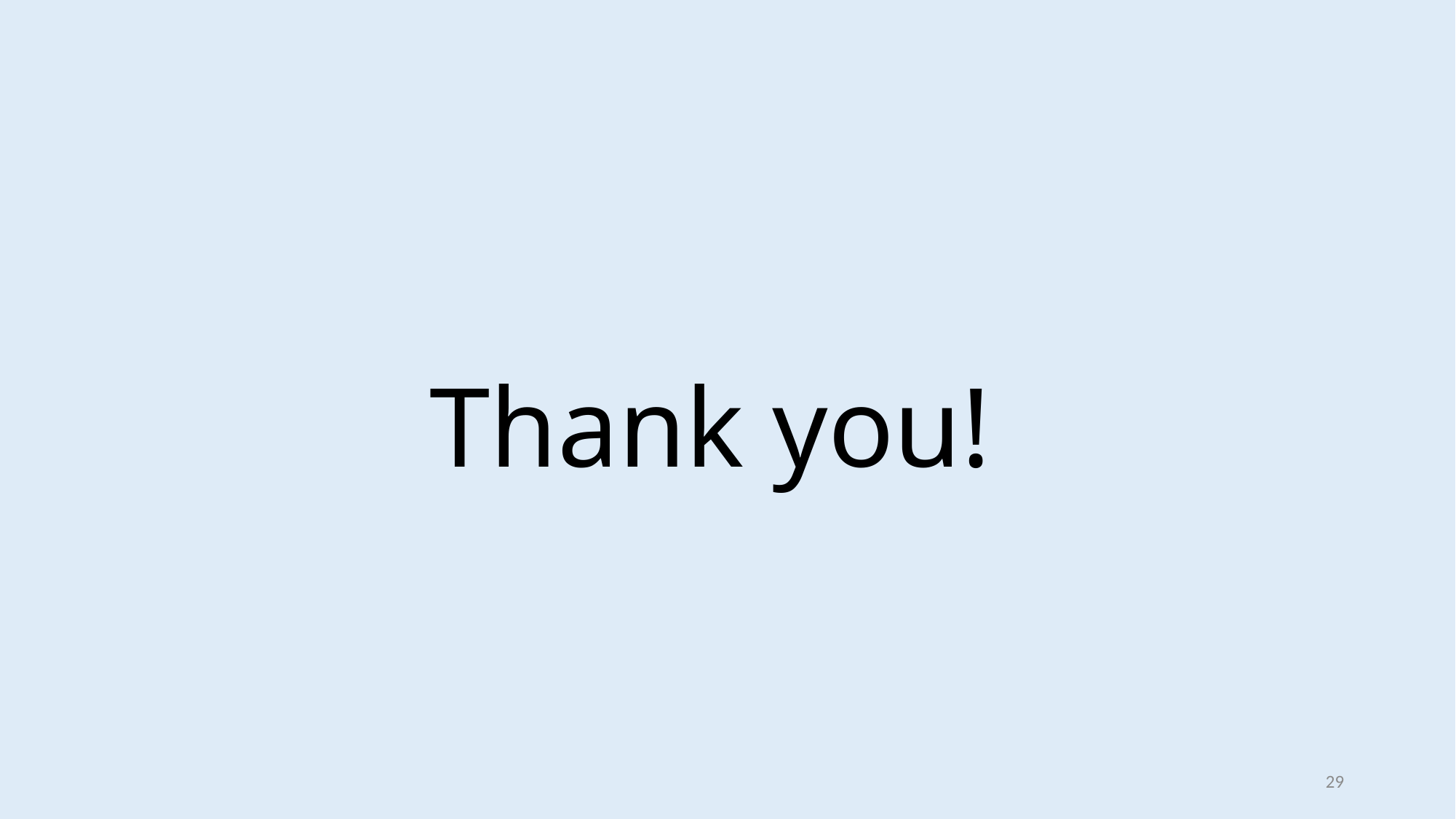

Thank you!
29

## Slide 30
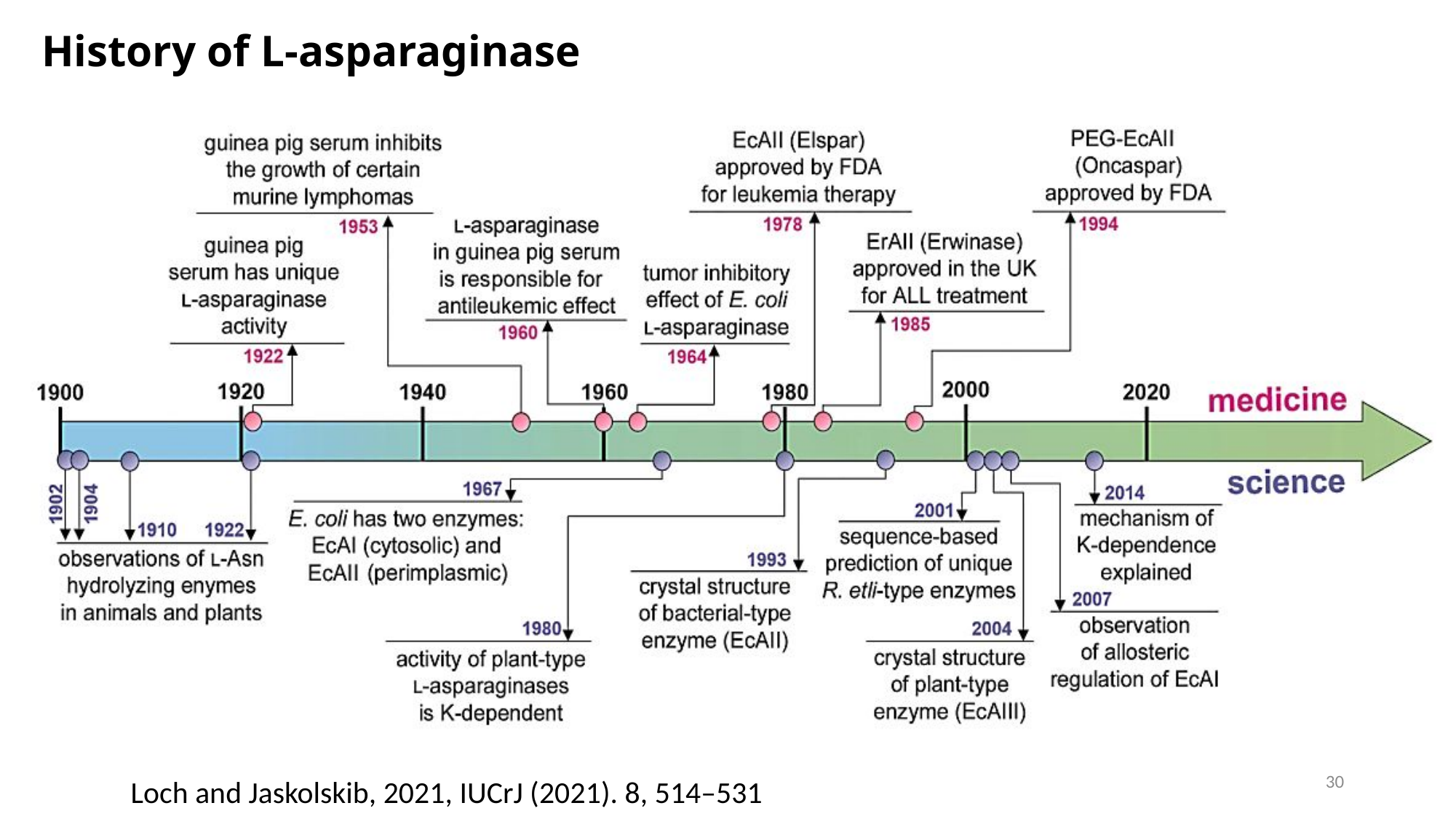

# History of L-asparaginase
30
Loch and Jaskolskib, 2021, IUCrJ (2021). 8, 514–531
